# MicroRNA-142-3p Overcomes Drug Resistance in Hepatocellular Carcinoma by Targeting YES1 and TWF1

**DOI:** 10.3390/ijms26094161

**Published:** 2025-04-27

**Authors:** Khadijeh Mahboobnia, Tasnuva D. Kabir, Rui Hou, Peiwen Liu, Alistair Forrest, Dianne J. Beveridge, Kirsty L. Richardson, Lisa M. Stuart, George C. Yeoh, Peter J. Leedman

**Affiliations:** 1 Harry Perkins Institute of Medical Research, QEII Medical Centre, 6 Verdun St, Nedlands, Perth, WA 6009, Australia; 22997944@student.uwa.edu.au (K.M.); tasnuva.kabir@perkins.org.au (T.D.K.); rui.hou@uwa.edu.au (R.H.); peiwen.liu@perkins.org.au (P.L.); alistair.forrest@perkins.org.au (A.F.); dianne.beveridge@perkins.org.au (D.J.B.); kirsty.richardson@perkins.org.au (K.L.R.); lisa.stuart@perkins.org.au (L.M.S.); george.yeoh@uwa.edu.au (G.C.Y.); 2 Centre for Medical Research, The University of Western Australia, Perth, WA 6009, Australia; 3 School of Molecular Sciences, The University of Western Australia, Perth, WA 6009, Australia

**Keywords:** hepatocellular carcinoma, miR-142-3p, drug resistance, YES1, TWF1, tyrosine kinase inhibitors

## Abstract

Resistance to tyrosine kinase inhibitors (TKIs, e.g., sorafenib and lenvatinib) presents a significant hurdle for hepatocellular carcinoma (HCC) treatment, underscoring the need to decipher the underlying mechanisms for improved therapeutic strategies. MicroRNAs (miRNAs) have emerged as critical modulators in HCC progression and TKI resistance. In this study, we report a positive correlation between the expression levels of a tumor suppressor miRNA, miR-142-3p, and increased sensitivity to sorafenib and lenvatinib, supported by clinical data from the BIOSTORM HCC cohort. Overexpression of miR-142-3p in TKI-resistant HCC cells significantly inhibited proliferation and colony formation, induced apoptosis, increased cell cycle arrest at the G2 phase, and reduced migration and invasion by reversing epithelial–mesenchymal transition. Notably, combining miR-142-3p with lenvatinib synergistically inhibited growth in both inherent and acquired TKI-resistant HCC cells by modulating critical signaling pathways, including STAT3, PI3K/AKT, MAPK, YAP1, and by impeding autophagic influx. RNA-sequencing of a TKI-resistant HCC cell line ± miR-142-3p overexpression identified YES1 and TWF1 as direct downstream target genes of miR-142-3p, both of which are key genes associated with drug resistance in HCC. Small interfering RNA (siRNA)-mediated knockdown of these genes mirrored the antitumor effects of miR-142-3p and enhanced TKI sensitivity, with YES1 knockdown decreasing YAP1 phosphorylation, and TWF1 knockdown inhibiting autophagy. Collectively, these findings indicate that restoring miR-142-3p expression or targeting its downstream effectors YES1 and TWF1 offers a promising strategy to overcome drug resistance and improve therapeutic outcome in HCC.

## 1. Introduction

Hepatocellular carcinoma (HCC) is a highly heterogeneous malignancy that accounts for 75–85% of human liver cancers and poses a significant public health challenge worldwide [1,2]. The primary factors contributing to HCC include cirrhosis caused by viral hepatitis (in particular hepatitis B virus (HBV) and hepatitis C virus (HCV)), excessive alcohol consumption, and non-alcoholic fatty liver disease [3]. Despite advances in diagnostic and therapeutic options, the prognosis for HCC remains poor, with a five-year survival rate of only 10–20% [3,4]. Treatment modalities, including receptor tyrosine kinase inhibitors (TKIs), such as sorafenib and lenvatinib, as well as a combination of immunotherapy and anti-angiogenesis therapy, including atezolizumab and bevacizumab, offer minimal survival benefit, and their efficacy is often limited due to the development of resistance [4]. Therapeutic resistance arises from multiple factors, including altered drug efflux, disrupted apoptotic pathways, remodeling of the tumor microenvironment, immune cell polarization that favors tumor survival, and changes in extracellular matrix components, as well as the acquisition of stem cell-like properties by cancer cells [4,5]. These alterations enable tumor cells to evade therapeutic effects and contribute to a more aggressive cancer phenotype [5]. These challenges emphasize the urgent need for innovative therapeutic strategies to enhance clinical outcomes for HCC patients.

MicroRNAs (miRNAs/miRs) are short non-coding RNAs (~22 nucleotides) that play a critical role in regulating post-transcriptional gene expression, thereby influencing various biological processes, including cell proliferation, differentiation, and apoptosis [6]. This regulatory mechanism involves binding of the miRNA to a specific seed sequence in the 3′-UTR of the target mRNA, facilitated by the RNA-induced silencing complex [6]. MicroRNAs can function as tumor suppressors or oncogenes in carcinogenesis, and can significantly impact essential cancer hallmarks [7]. Aberrant miRNA expression profiles in HCC can contribute to tumor growth, metastatic potential, and response to therapies, making them potential biomarkers for diagnosis and prognosis, as well as targets for therapeutic intervention [8,9], with studies showing promising results in preclinical models [10]. The identification of miRNAs and their specific roles in cancer progression and therapy response may provide a molecular roadmap for the development of more effective treatment strategies for advanced HCC [8]. The therapeutic potential of miRNA mimics or inhibitors (anti-miRs) represents a cutting-edge approach for precision therapy [9,11].

In the current study, we used an in silico approach to identify miR-142-3p as a miRNA with therapeutic potential in HCC. Overexpression of miR-142-3p resulted in a reduction of HCC cell line growth, invasion, and migration. An RNA-sequencing analysis identified *YES1* and *TWF1* as target genes of miR-142-3p in TKI-resistant HCC cell lines. Additionally, we showed that both miR-142-3p overexpression or the knockdown of its gene targets acted synergistically with TKIs, such as sorafenib or lenvatinib, to enhance therapeutic efficacy. These data collectively support the notion that therapeutically miR-142-3p mimics or drugs inhibiting its target genes could be effective treatment options for HCC patients and provide a survival benefit.

## 2. Results

### 2.1. Identification of miR-142-3p as a Potential Tumor Suppressor miRNA in HCC

**Figure 1:**
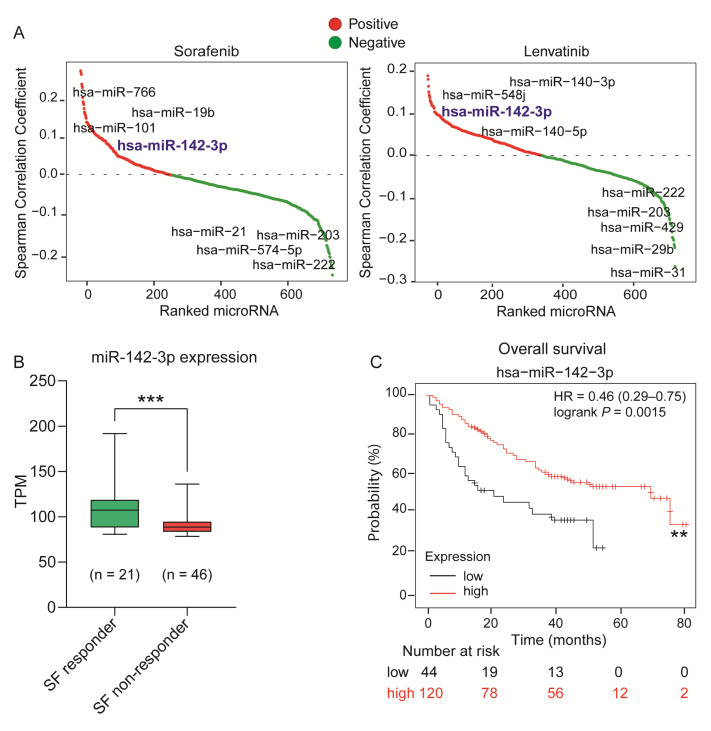
**Identification of miR-142-3p as a potential tumor suppressor miRNA in HCC.** (**A**) Waterfall plot depicting the correlation between basal miRNA expression and TKI (sorafenib/lenvatinib) sensitivity in human cancer cell lines (data source: Cancer Cell Encyclopedia and Cancer Therapeutic Response Portal). (**B**) RNA-sequencing data of transcript per million (TPM) values of miR-142-3p expression in the sorafenib responder vs. non-responder group (BIOSTORM HCC cohort) from GSE109211 in the GEO datasets. Error bars = SD; *** *p* ≤ 0.001. (**C**) Kaplan–Meier plot of 166 HCC patients at different stages of the disease to determine the prognostic value of miR-142-3p expression. The *p*-value was calculated using the log-rank test; ** *p* = 0.0015. Abbreviations: SF: sorafenib; HR: hazard ratio.

To identify miRNAs that may have specific roles in HCC progression and therapy response, we initially employed a bioinformatics approach to explore the correlation between miRNA expression profiles and TKI sensitivity (specifically sorafenib, lenvatinib, and cabozantinib) in 791 human cancer cell lines. A positive correlation coefficient indicated that higher basal expression of a miRNA corresponded to an increased TKI sensitivity; whereas a negative correlation coefficient indicated that elevated basal levels of a miRNA were associated with reduced response to TKIs. We found miR-142-3p expression to be positively correlated with sensitivity to all three TKIs suggesting that cancer cells with higher levels of miR-142-3p are more responsive to sorafenib, lenvatinib, and cabozantinib treatment (Figure 1A/Figure S1A).

To explore the clinical relevance of this association between miR-142-3p expression and TKI sensitivity, both the miRNA and mRNA expression data from HCC patients in the BIOSTORM cohort of the STORM trial (GSE109211 in the GEO datasets) were analyzed. Of the 140 patients in the trial, 67 received treatment with sorafenib, with 21 of these patients classified as responders and 46 as non-responders to sorafenib, with the sorafenib responder group having a higher baseline expression level of miR-142-3p (Figure 1B). Additionally, a Kaplan–Meier analysis of 166 liver cancer patients using the KM Plotter demonstrated that those with elevated miR-142-3p expression had better overall survival outcomes (Figure 1C). These findings indicate a plausible role of miR-142-3p in enhancing TKI sensitivity.

### 2.2. MicroRNA-142-3p Inhibits HCC Cell Survival by Regulating Cell Cycle and Apoptosis

**Figure 2:**
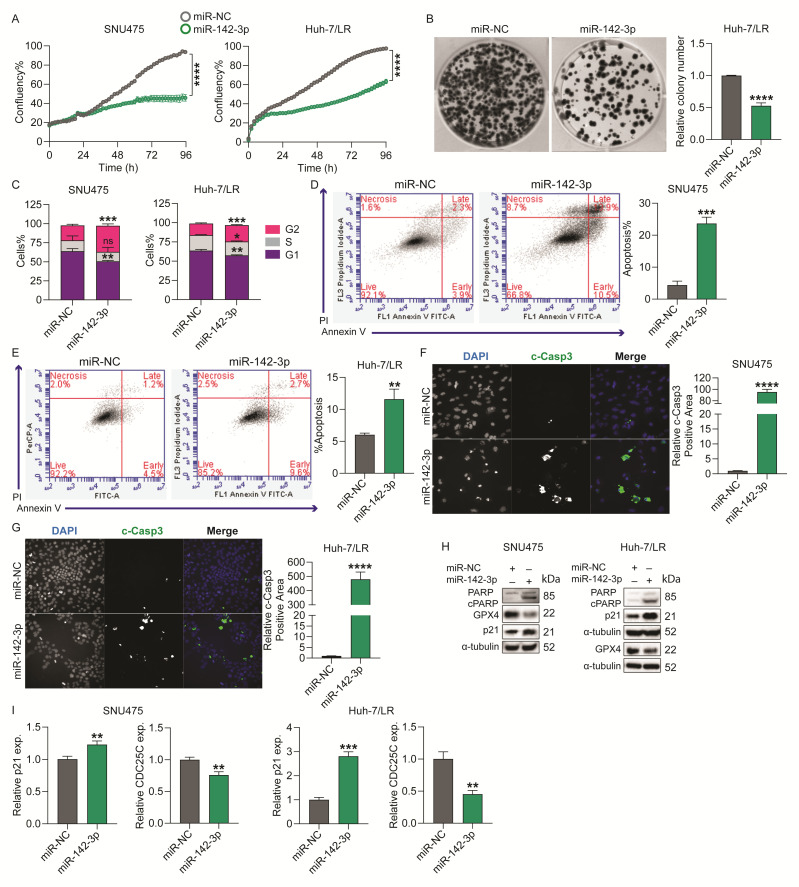
**Functional effects of miR-142-3p on proliferation and survival of HCC cells.** SNU475 and Huh-7/LR cells were transfected with a 10 nM miR-142-3p mimic or miR-NC and assayed as follows: (**A**) Incucyte proliferation assay: time-lapse imaging was conducted at 2-h intervals continuously for up to 96 h. (**B**) Huh-7/LR cell colony formation capacity 14 days post-transfection. (**C**) Flow cytometric analysis of the cell cycle distribution in HCC cells 72 h post-transfection, following propidium iodide staining. (**D**,**E**) The percentage of apoptotic cells was assessed using Annexin V-FITC/propidium iodide staining followed by flow cytometry, with measurements taken 96 h post-transfection for SNU475 cells (**D**) and 72 h post-transfection for Huh-7/LR cells (**E**); bar graphs represent the percentage of apoptotic cells. (**F**,**G**) Immunofluorescence staining of SNU475 (**F**) and Huh-7/LR (**G**) cells with DAPI and cleaved-caspase 3 (c-Casp3) 96 h after transfection. The magnification is 20× and the image analysis was performed using Thermo Fisher Scientific HCS Studio 2.0 Cell Analysis Software on the CellInsight CX7 High Content Analysis System. (**H**) Western blot analysis of cleaved PARP (cPARP), glutathione peroxidase 4 (GPX4), and p21 protein expression levels 96 h post-transfection. α-tubulin was used as a loading control. (**I**) RT-qPCR analysis of genes related to cell cycle arrest (p21) and the G2/M transition (CDC25C) after 72 h of transfection. Data were analyzed using the 2^−ΔΔCt^ method with GAPDH as the reference gene and expressed relative to miR-NC. Error bars = SEM (for **A**) and = SD (for **B**–**I**); n = 3. Significance levels are indicated as follows: ns = not significant, * *p* ≤ 0.05, ** *p* ≤ 0.01, *** *p* ≤ 0.001, **** *p* ≤ 0.0001.

To assess the functional effects of miR-142-3p on HCC cell growth, we transiently overexpressed miR-142-3p in various HCC cell lines, including inherently TKI-resistant SNU475 and PLC/PRF/5, acquired sorafenib-resistant Huh-7/SR1, and acquired lenvatinib-resistant Huh-7/LR cells. Based on Incucyte time-lapse imaging, miR-142-3p significantly inhibited cell proliferation in all four cell lines (Figure 2A/Figure S2A), and markedly reduced the colony formation capacity of Huh-7/LR and PLC/PRF/5 cells (Figure 2B/Figure S2B).

To explore the mechanisms of miR-142-3p overexpression on cell proliferation, we next investigated miR-142-3p’s effect on cell cycle, apoptosis, and ferroptosis. A cell cycle analysis showed overexpression of miR-142-3p stimulated arrest at the G2/M phase in SNU475, Huh-7/LR, and Huh-7/SR1, and at the G1/S phase in PLC/PRF/5 cells compared to the miR-NC control (Figure 2C/Figure S2C), and significantly induced apoptosis in all cell lines (Figure 2D,E/Figure S2D,E). The induction of apoptosis was further validated using immunofluorescence staining for c-Casp3 and western blot for cleaved poly (ADP-ribose) polymerase (c-PARP), both of which are well-established markers of apoptosis [12,13]. We found miR-142-3p overexpression increased the c-Casp3 positive stained area in SNU475, Huh-7/LR, and in PLC/PRF/5 cells (Figure 2F,G/Figure S2F), and stimulated expression of c-PARP protein levels in SNU475 and Huh-7/LR cells (Figure 2H). We also assessed the effect of miR-142-3p overexpression on ferroptosis, a form of cell death that is characterized by iron dependency and lipid peroxidation, and has emerged as a promising therapeutic target in HCC [14], by examining the expression of glutathione peroxidase 4 (GPX4), a key factor in inhibiting ferroptosis, and found its levels to be significantly reduced with miR-142-3p overexpression (Figure 2H).

Given that miR-142-3p stimulated cell cycle arrest at G2/M, a characteristic displayed by drugs causing apoptosis, we examined its impact on the expression of the G2/M checkpoint proteins CDC25C and p21 in our HCC cell lines. CDC25C is a dual phosphatase which functions at the G2/M checkpoint, where it dephosphorylates and activates cyclin-dependent kinase 1 (CDK1), an essential step for G2 to M transition, initiating mitosis [15], and p21 induces G2/M arrest by inhibiting cyclin B/CDK1 complexes, preventing the cell from entering mitosis in response to DNA damage [16]. Corroborating with our previous findings, miR-142-3p overexpression significantly increased expression of p21 at both protein and mRNA levels in SNU475 and Huh-7/LR cells (Figure 2H,I/Figure S2G), and reduced the expression of CDC25C in SNU475, Huh-7/LR, and Huh-7/SR1 cells (Figure 2I/Figure S2G), implying that miR-142-3p overexpressing HCC cells are less likely to enter mitosis and rather progress towards apoptosis. Taken together, these results suggest that miR-142-3p inhibits HCC cell proliferation via halting cell cycle progression and inducing apoptosis.

To explore if the effects of miR-142-3p overexpression are opposite to those when miR-142-3p is depleted from cells, we initially assessed a panel of HCC cell lines to establish which line had the highest basal expression of miR-142-3p. SNU423 cells endogenously expressed the highest level of miR-142-3p compared to the other HCC cell lines (Figure S1B), and was selected as the cell line to explore the effects of miR-142-3p depletion. SNU423 cells were transiently transfected with an miR-142-3p mimic or an anti-miR-142-3p (and relevant negative controls), and miR-142-3p overexpression resulted in a marked decrease in proliferation compared to miR-142-3p knockdown cells. (Figure S2H). Collectively, these data indicate that miR-142-3p is a tumor suppressor in HCC cell lines.

### 2.3. Overexpression of miR-142-3p Suppresses HCC Cell Chemotaxis and Invasion

**Figure 3:**
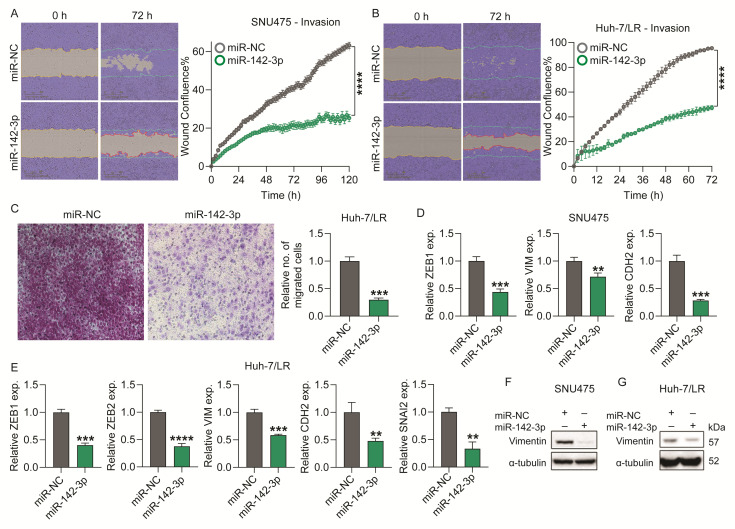
**Functional impact of miR-142-3p on HCC cell motility.** SNU475 and Huh-7/LR cells were transfected with 10 nM miR-142-3p or miR-NC and assayed as follows: (**A**,**B**) Wound healing assays were conducted in the presence of Matrigel to assess the invasion ability of SNU475 (**A**) and Huh-7/LR. (**B**). Wound confluence (%) within the scratch area was calculated based on phase-contrast images using the Incucyte ZOOM software, version 2018A (Essen BioScience). Blue lines represent the initial scratch boundaries, and red lines indicate the extent of cell invasion into the scratch area. (**C**) Transwell chemotaxis assay was used to examine Huh-7/LR cell chemotaxis 48 h after transfection. The images were captured at a magnification of 20× using an Olympus IX-71 Inverted Fluorescence Microscope, with a minimum of four fields per well. (**D**,**E**) RT-qPCR analysis of EMT markers in miR-142-3p transfected cells compared to miR-NC control. Data were analyzed using the 2^−ΔΔCt^ method with GAPDH as the reference gene. (**F**,**G**) Western blot analysis of vimentin protein expression level after 96 h of transfection. α-tubulin was used as an internal control (housekeeping protein). Error bars = SEM (for **A**,**B**) and = SD (for **C**–**E**); n = 3. Significance levels are indicated as follows: ** *p* ≤ 0.01, *** *p* ≤ 0.001, **** *p* ≤ 0.0001.

As previous studies have shown, miR-142-3p suppresses epithelial–mesenchymal transition (EMT) and metastasis in breast cancer models [17], and EMT and invasion in colorectal cancer cells [18]. We investigated the effects of miR-142-3p on invasion and chemotaxis in TKI-resistant HCC cell lines. Aligning with previous studies, we saw that miR-142-3p overexpression significantly inhibited SNU475, Huh-7/LR, PLC/PRF/5, and Huh-7/SR1 cell invasion in an Incucyte wound healing assay compared to the control (Figure 3A,B/Figure S3A,B). Furthermore, a substantial reduction in Huh-7/LR cell chemotaxis was observed in a 2D transwell migration assay (Figure 3C).

To assess whether miR-142-3p could reverse EMT, we examined the expression levels of several EMT-related genes following miR-142-3p overexpression in SNU475, Huh-7/LR, and Huh-7/SR1 cells. MicroRNA-142-3p significantly downregulated *ZEB1*, *VIM* (vimentin), and *CDH2* (N-cadherin) mRNA levels in each cell line (Figure 3D,E/Figure S3C). We also observed a significant reduction in the expression of *ZEB2* in Huh-7/LR cells (Figure 3E) and *SNAI2* (Slug) in Huh-7/SR1 cells (Figure S3C). Furthermore, miR-142-3p overexpression decreased vimentin at the protein level in SNU475 and Huh-7/LR cells (Figure 3F,G). Taken together, these findings support an inhibitory impact of miR-142-3p on HCC cell motility and EMT.

### 2.4. Identification of miR-142-3p Downstream Gene Targets and Molecular Pathways

**Figure 4:**
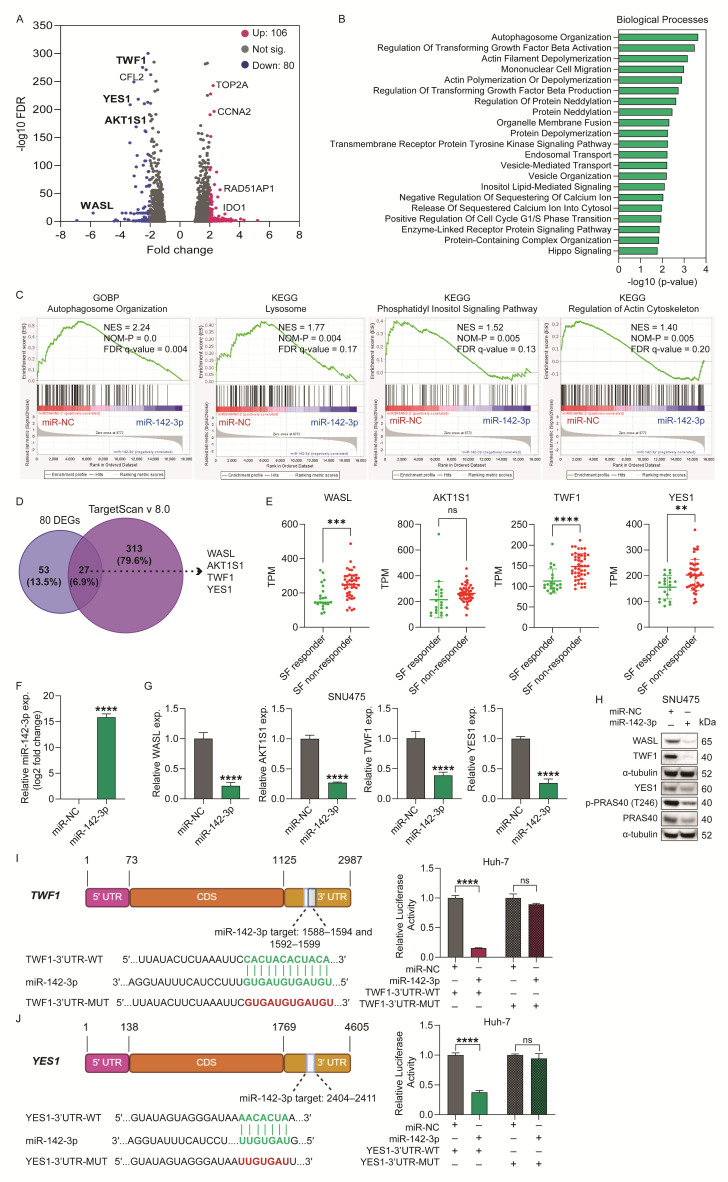
**Mechanistic pathway analysis and identification of miR-142-3p targets.** (**A**) Volcano plot of differentially expressed genes between SNU475 cells transfected ± 10nM miR-142-3p mimic or miR-NC control for 24 h, using >2.0-fold change and an FDR of *p* < 0.05. The 106 transcripts that were identified as significantly upregulated are colored in pink, and the 80 significantly downregulated transcripts are shown in blue. Gray dots indicate no significance (Not sig.) difference between genes expression. (**B**) Biological processes affected by the 80 downregulated genes using the Enrichr tool. (**C**) Representative enrichment plots were generated using the GSEA tool to analyze the downregulated genes from the entire RNA-seq data (FDR < 25%, *p* < 0.01). The green line represents the running enrichment score (ES) for the gene set, and the black vertical bars indicate the positions of the individual genes from the gene set within the ranked list. The bottom gray panel displays the rank order of all differentially expressed genes. (**D**) Venn diagram of the overlap between the 80 genes downregulated by miR-142-3p in SNU475 cells from the RNA-seq, and 340 predicted target genes for miR-142-3p on TargetScan (v.8.0) with a cumulative weighted context++ score of <−0.25 and at least one seed sequence. (**E**) TPM values of the expression profiles of miR-142-3p target genes were analyzed in sorafenib responders versus non-responders from the BIOSTORM HCC cohort, using data from GSE109211 in the GEO database. SNU475 cells were transfected with 10 nM miR-142-3p or miR-NC and assayed as follows: (**F**) miR-142-3p TaqMan microRNA assay with RT-qPCR, showing log2 fold-change normalized to U6 snRNA at 24 h post-transfection. (**G**) RT-qPCR analysis of the impact of miR-142-3p overexpression on the mRNA expression levels of the four identified target genes at 24 h post-transfection. Data were analyzed using the 2^−ΔΔCt^ method, with GAPDH as the reference gene. (**H**) Western blot of gene target protein expression at 96 h post-transfection. PRAS40 is encoded by the AKT1S1 gene. α-tubulin was used as an internal control (housekeeping protein). (**I**,**J**) Luciferase reporter assay in Huh-7 cells 24 h post-co-transfection with 25 nM miR-142-3p or miR-NC and 500 ng of the reporter plasmids; TWF1 (**I**) and YES1 (**J**). A schematic representation of the wild-type (WT) and mutant (MUT) 3′-UTR vectors illustrating miRNA-binding sites is presented. The wild-type binding sites are indicated in green, and the mutated sites are highlighted in red. Luciferase activity is presented as relative to miR-NC. Error bars = SD; n = 3; ns = not significant, ** *p* < 0.01, *** *p* < 0.001, and **** *p* < 0.0001. Abbreviations: GSEA: gene set enrichment analysis; GOBP: gene ontology biological process; KEGG: Kyoto Encyclopedia of Genes and Genomes; NES: normalized enrichment score; NOM-P: nominal p-value; FDR: false discovery rate; TPM: transcripts per million; SF: sorafenib; UTR: untranslated region; CDS: coding sequence.

To identify downstream gene targets and the molecular pathways affected by miR-142-3p overexpression, we transiently transfected the inherently TKI-resistant SNU475 cell line (which has very low endogenous levels of miR-142-3p (Table S1) and possesses aggressive tumorigenic properties) with miR-142-3p or miR-NC, and performed RNA-sequencing (RNA-seq) on these cells. Using a threshold of a 5% false discovery rate (FDR < 0.05) and at least a two-fold change in expression, we identified 186 differentially expressed genes (DEGs) between the two groups; 80 genes were downregulated and 106 genes were upregulated in response to miR-142-3p overexpression (Figure 4A, Table S3).

Notably, among the upregulated genes, TOP2A and CCNA2 were significantly increased following miR-142-3p overexpression (Figure 4A). While both genes have been associated with poor prognosis in liver cancer, as supported by TCGA survival data and previously published studies [19,20], this upregulation is likely an indirect effect. It is well-established that miRNAs can influence gene expression networks beyond their direct targets—often by downregulating transcriptional repressors or modulators, thereby indirectly activating other genes [21]. Furthermore, miRNAs may have context-dependent effects and can modulate both oncogenic and tumor-suppressive pathways [22]. In our study, despite the upregulation of TOP2A and CCNA2, miR-142-3p exhibited an overall tumor-suppressive phenotype, as evidenced by the inhibition of proliferation, survival, and migration pathways. These findings suggest that the net effect of miR-142-3p remains anti-tumorigenic.

Given that miRNAs modulate gene expression through gene silencing, we focused on the downregulated genes and conducted a pathway analysis on the entire downregulated gene set using Gene Set Enrichment Analysis (GSEA v 4.1.10), and on the 80 significantly downregulated genes using the Enrichr tool (https://maayanlab.cloud/Enrichr/). Figure 4B illustrates the Enrichr biological processes (ranked by *p*-value) in which the genes downregulated by miR-142-3p overexpression are most significantly enriched and include autophagosome organization, actin filament depolymerization, cell migration, organelle membrane fusion, the transmembrane receptor protein tyrosine kinase signaling, and Hippo signaling pathways. Figure 4C displays enrichment plots from the Gene Ontology Biological Processes (GOBP) and KEGG pathway analyses, demonstrating that miR-142-3p overexpression leads to downregulation of gene sets related to autophagosome organization, lysosomal function, phosphatidylinositol signaling, and regulation of the actin cytoskeleton. Taken together these pathway analyses support the role of miR-142-3p in modulating key cellular processes including autophagy, cytoskeletal dynamics, and signaling pathways that may contribute to its effects on cell invasion and tumorigenicity in the TKI-resistant SNU475 cell line.

Next, we conducted an intersection analysis between the 80 downregulated genes identified in the RNA-seq data and the 340 predicted targets of miR-142-3p obtained from the TargetScan database v.8.0 (https://www.targetscan.org), with the selected targets having a cumulative weighted context++ score of < −0.25 and containing at least one miR-142-3p seed sequence. As shown by the Venn diagram in Figure 4D, 27 genes overlapped between the two groups and included WASL, AKT1S1, TWF1, and YES1. These genes are involved in diverse cellular functions relevant to cancer biology: WASL (Wiskott–Aldrich syndrome like) regulates actin cytoskeleton remodeling and cell motility [23]; AKT1S1 (also known as PRAS40) is a substrate of AKT and modulates mTORC1 activity [24]; TWF1 (twinfilin-1) is an actin-binding protein that controls cytoskeletal dynamics and contributes to EMT [25]; and YES1 is an Src family tyrosine kinase involved in survival signaling, cell proliferation, and drug resistance [26]. We evaluated the mRNA expression levels of these four genes in the sorafenib treatment cohort of the BIOSTORM HCC trial. The expression levels of all four genes were higher in the sorafenib non-responder group (Figure 4E), indicating a potential association between these genes and TKI resistance. Following validation of miR-142-3p overexpression (Figure 4F/Figure S4A), RT-qPCR and western blot analysis demonstrated that overexpression of miR-142-3p led to a substantial downregulation of all four genes at both the mRNA and protein level in multiple HCC cell lines (Figure 4G,H/Figure S4B–D). Further, to validate the regulatory relationship between miR-142-3p and its candidate target genes, we performed both gain- and loss-of-function experiments in SNU423 cells. Successful overexpression and knockdown of miR-142-3p were confirmed by RT-qPCR (Figure S4E). As shown in Figure S4F, miR-142-3p overexpression significantly reduced the mRNA levels of WASL, AKT1S1, TWF1, and YES1, while miR-142-3p depletion, using a specific anti-miR, significantly rescued and upregulated the expression of all four genes compared to anti-miR-NC controls. These findings further support that miR-142-3p negatively regulates these genes at the transcript level.

To guide our selection of candidates for further functional validation, we reviewed all 27 overlapping genes (Figure 4D) for their known or potential association with chemoresistance, as detailed in Table S4. Based on this evaluation, we analyzed the expression of nine additional genes—ASH1L, ITGB8, PLCB1, VAMP3, RARG, NR2F6, CFL2, LRRC32, and MRFAP1—using the BIOSTORM HCC cohort data, comparing sorafenib responders versus non-responders. As shown in Figure S4G, several genes, including ASH1L, NR2F6, LRRC32, and MRFAP1, were significantly upregulated in non-responders. However, these genes are more commonly associated with immune modulation, tumor microenvironment (TME) remodeling, or general cell cycle regulation, rather than the direct tumor cell–intrinsic mechanisms of drug resistance [27,28,29,30]. Since our study focused on the direct regulatory effects of miR-142-3p within HCC cells, we selected YES1 and TWF1 for in-depth investigation based on their established involvement in survival signaling, autophagy, and cytoskeletal remodeling—key processes that underlie adaptive drug resistance and escape mechanisms in multiple cancers [25,31,32,33].

A TargetScan analysis revealed that TWF1 contains two binding sites for miR-142-3p within its 3′-UTR (positions 1588–1594 and 1592–1599), while YES1 has a single binding site at position 2404–2411 in its 3′-UTR (Figure 4I,J). To verify if these genes are direct targets of miR-142-3p, Huh-7 cells were co-transfected with pEZX-MT06 luciferase reporter constructs containing either the wild-type or mutated 3′-UTRs of TWF1 and YES1, along with either an miR-142-3p mimic or an miR-NC control. Our results indicated that miR-142-3p significantly reduced luciferase activity in cells transfected with the wild-type plasmids compared to miR-NC controls (Figure 4I,J). This reduction in luciferase activity was restored in cells transfected with the mutant TWF1 or YES1 3′-UTR constructs (Figure 4I,J). Taken together these data support TWF1 and YES1 as direct targets of miR-142-3p, warranting further investigation into their potential as therapeutic targets for HCC treatment.

### 2.5. YES1 Knockdown Reduced Survival, Colony Formation and Migration/Invasion of TKI-Resistant HCC Cells In Vitro

YES proto-oncogene 1 (YES1) is a member of the SRC family of kinases (SFK) and plays a significant role in various cellular processes that are critical in tumorigenesis [31]. YES1 amplification has been associated with resistance to chemotherapeutic drugs in human malignancies, suggesting that YES1 signaling is a potential target for overcoming drug resistance [31]. Therefore, we investigated the functional effects of YES1 in TKI-resistant HCC cell lines.

**Figure 5:**
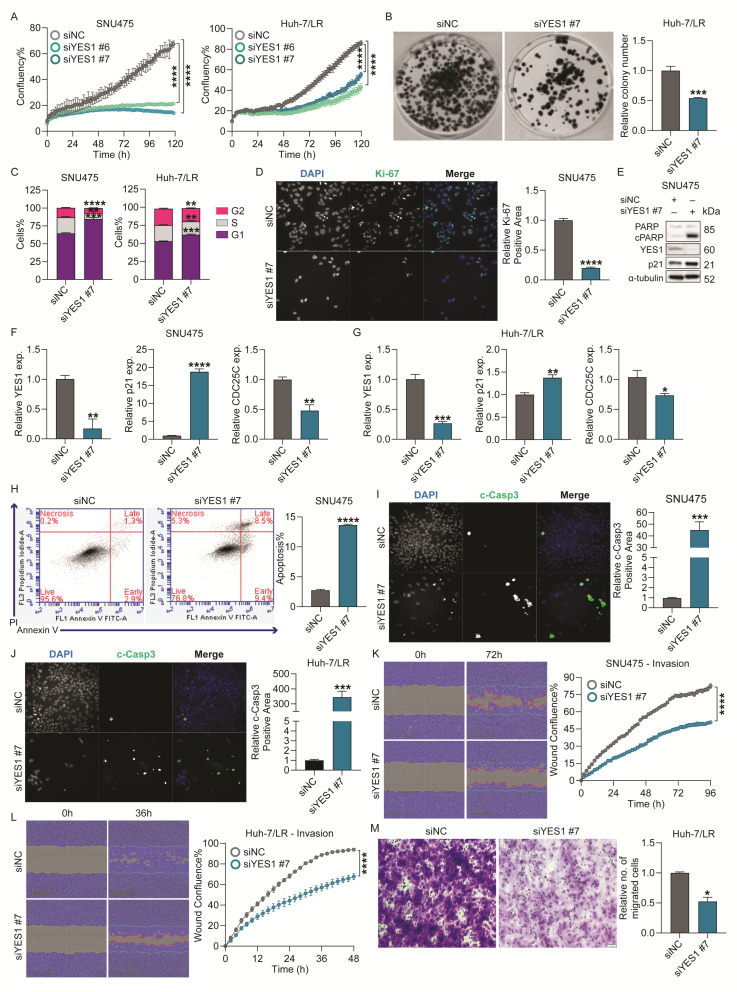
**The effect of YES1 knockdown on HCC cell survival and motility.** SNU475 or Huh-7/LR cells were transiently transfected with 10 nM YES1 specific siRNAs siYES1 #6 or #7, or a siNC control and assayed as follows. The “#” symbol indicates distinct siRNAs targeting different sequences of the YES1 mRNA transcript, with the numbers referring to individual products supplied by the manufacturer. (**A**) Incucyte proliferation assay with time-lapse imaging conducted at 2-h intervals continuously for up to 120 h. (**B**) Effect of YES1 depletion on colony formation capacity of Huh-7/LR cells 14 days post-transfection. (**C**) Flow cytometric analysis of the cell cycle distribution in SNU475 and Huh-7/LR cells at 72 h post-transfection, following propidium iodide staining. (**D**) Immunofluorescence analysis of Ki-67 expression 72 h post-transfection of SNU475 cells. The magnification is 20× and the image analysis was performed using Thermo Fisher Scientific HCS Studio 2.0 Cell Analysis Software on the CellInsight CX7 High Content Analysis System. (**E**) Western blot analysis of cleaved PARP, YES1, and p21 protein expression level after 96 h of transfection of SNU475 cells. α-tubulin was used as an internal control (housekeeping protein). (**F**,**G**) RT-qPCR analysis of SNU475 (**F**) and Huh-7/LR (**G**) cells to validate YES1 gene knockdown and genes related to cell cycle arrest (p21) and G2/M transition (CDC25C) 72 h post-transfection; data were analyzed using the 2^-ΔΔCt^ method with GAPDH as the reference gene. (**H**) YES1 downregulation-associated apoptosis was assessed by Annexin V-FITC/propidium iodide staining and flow cytometry 72 h post-transfection. (**I**,**J**) Immunofluorescence staining of cleaved caspase 3 (c-Casp3) 72 h post-transfection of SNU475 **(I**) and Huh-7/LR **(J**) cells; imagining parameters are as per above (Figure 5D). (**K**,**L**) The effect of YES1 knockdown on the invasive ability of SNU475 (**K**) and Huh-7/LR (**L**) cells was evaluated using a wound healing assay in the presence of Matrigel; cell invasion was quantified by calculating the percentage of wound confluence within the scratch area based on phase-contrast images analyzed using Incucyte ZOOM software, version 2018A (Essen BioScience); blue lines indicate the initial scratch boundary, and red lines indicate the extent of cell invasion into the scratch area. (**M**) Transwell chemotaxis assay. The Huh-7/LR cell chemotaxis ability was assessed 48 h post-transfection. The magnification is 20× and images were captured using an Olympus IX-71 Inverted Fluorescence Microscope, with a minimum of four fields per well. Error bars = SEM (**A**,**K**,**L**), or SD (**B**–**J**); n = 3. Significance levels are indicated as follows: * *p* ≤ 0.05, ** *p* ≤ 0.01, *** *p* ≤ 0.001, and **** *p* ≤ 0.0001.

To explore the role of YES1 in HCC we used specific siRNAs (siYES1 #6 and siYES1 #7) to transiently knockdown YES1 in inherent (SNU475, PLC/PRF/5) and acquired (Huh-7/LR, Huh-7/SR1) TKI-resistant HCC cells, and observed a significant inhibition of proliferation in all cell lines with YES1 reduction (Figure 5A/Figure S5C). Consistently, the colony formation capacity of Huh-7/LR and PLC/PRF/5 cells was attenuated after YES1 depletion (Figure 5B/Figure S5D). A cell cycle analysis revealed siYES1 #7 induced a G1/S arrest in SNU475, Huh-7/LR, and Huh-7/SR1 cells compared to siNC control (Figure 5C/Figure S5E), which corresponded with a significant reduction in the number of Ki-67 positive nuclei (Figure 5D/Figure S5F). In PLC/PRF/5 cells, however, siYES1 #7 treatment stimulated arrest at G2/M phase of the cell cycle (Figure S5E). We also observed elevated levels of p21 (both mRNA and protein) and reduced levels of CDC25C (mRNA) in SNU475 and Huh-7/LR cells (Figure 5E–G), corroborating a defect in the cell cycle and progress toward apoptosis, as observed with miR-142-3p.

Evaluation of the effect of YES1 on cancer cell apoptosis demonstrated that its suppression significantly increased the percentage of both early and late apoptotic cell population in SNU475 and PLC/PRF/5 (Figure 5H/Figure S5G). This finding was further validated with immunofluorescence staining for c-Casp3 (Figure 5I,J/Figure S5H) and western blot for cleaved-PARP (Figure 5E), both of which were significantly upregulated by siYES1, confirming induction of apoptosis.

Next, we explored the role of YES1 in HCC cell migration and invasion. In a wound healing assay, YES1 knockdown significantly diminished the invasive capacity of both SNU475 and Huh-7/LR cells in Matrigel (Figure 5K,L), and reduced the migratory potential of PLC/PRF/5 cells (Figure S5I). We also assessed if YES1 inhibition affected chemotaxis of Huh-7/LR cells using a 2D transwell assay. As before, YES1 knockdown led to a notable reduction in the Huh-7/LR cells chemotaxis (Figure 5M). Collectively, these findings verify the role of YES1 in promoting survival, migration, and invasion of TKI resistant HCC cells.

### 2.6. TWF1 Knockdown Reduced Survival, Colony Formation, and Migration/Invasion Capabilities of TKI-Resistant HCC Cells In Vitro

Twinfilin-1 (TWF1) is an actin-binding protein primarily in the cytosol, crucial for managing actin dynamics by binding to and sequestering actin monomers [34,35]. This process influences the assembly and turnover of the actin cytoskeleton, essential for autophagy, as it aids in the formation and movement of autophagosomes [36,37]. The emerging literature highlights TWF1’s role in drug sensitivity and cancer progression, indicating its potential as a therapeutic target [33,38]. Therefore, we investigated the effects of TWF1 in TKI-resistant HCC cell lines.

**Figure 6:**
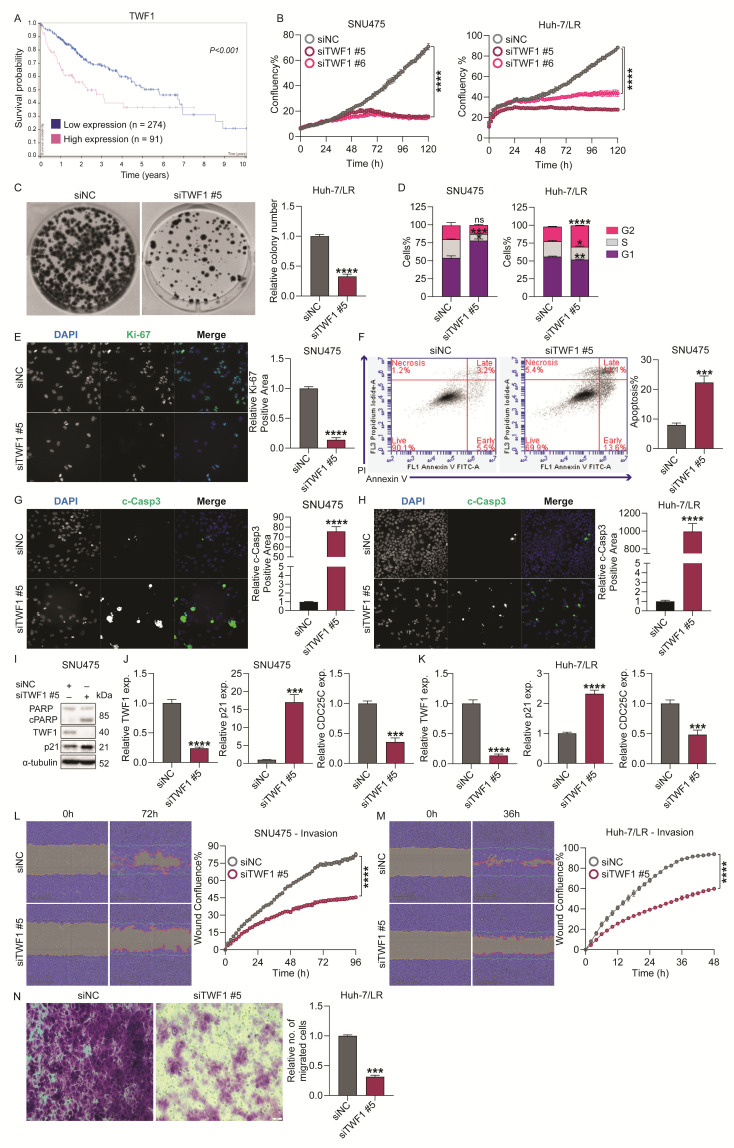
**Effects of TWF1 knockdown on HCC cell survival and motility.** (**A**) Kaplan–Meier survival analysis of 365 patients with HCC to determine the prognostic value of TWF1. Patients were categorized into low and high TWF1 expression groups based on data from the Human Protein Atlas (*p* < 0.001) (https://www.proteinatlas.org/ENSG00000151239-TWF1/pathology/liver+cancer). SNU475 or Huh-7/LR cells were transiently transfected with siRNAs to TWF1 (siTWF1 #5 or #6) or siNC control and assayed as follows. The “#” symbol indicates different siRNAs targeting distinct sequences within the TWF1 mRNA transcript; the numbers correspond to specific product identifiers provided by the supplier. (**B**) Incucyte proliferation assay using time-lapse imaging at 2-h intervals continuously for up to 120 h. (**C**) Effect of TWF1 knockdown on colony formation capacity of Huh-7/LR cells 14 days post-transfection. (**D**) Flow cytometric analysis of the cell cycle distribution in SNU475 and Huh-7/LR cells at 72 h post-transfection, following propidium iodide staining. (**E**) Immunofluorescence analysis of Ki-67 expression 72 h post-transfection of SNU475 cells. (**F**) TWF1 downregulation-associated apoptosis was assessed by Annexin V-FITC/propidium iodide staining and flow cytometry 72h after transfection. (**G**,**H**) Immunofluorescence staining of cleaved caspase 3 (c-Casp3) 72 h post-transfection in SNU475 (**G**) and Huh-7/LR (**H**) cells, to validate apoptosis induction. (**I**) Western blot analysis of cleaved PARP, TWF1, and p21 expression 96 h post-transfection of SNU475 cells. α-tubulin was used as an internal control (housekeeping protein). (**J**,**K**) RT-qPCR analysis of SNU475 (**J**) and Huh-7/LR (**K**) cells to validate TWF1 gene knockdown and genes related to cell cycle arrest (p21) and G2/M transition (CDC25C) 72 h after transfection; data were analyzed using the 2^−ΔΔCt^ method with GAPDH as the reference gene. (**L**,**M**) The effect of TWF1 knockdown on the invasive ability of SNU475 (**L**) and Huh-7/LR (**M**) cells was evaluated using a wound healing assay in the presence of Matrigel; cell invasion was quantified by calculating the percentage of wound confluence within the scratch area based on phase-contrast images analyzed using Incucyte ZOOM software, version 2018A (Essen BioScience). Blue lines indicate initial scratches, and red lines show the boundary of cells that have invaded into the scratch area.Data are presented as mean ± SEM. (**N**) Transwell chemotaxis assay. Huh-7/LR cell chemotaxis ability was assessed 48 h after transfection. The magnification is 20× and images were captured using an Olympus IX-71 Inverted Fluorescence Microscope, with a minimum of four fields per well. All immunofluorescence was with a 20× magnification, and the image analysis was performed using Thermo Fisher Scientific HCS Studio 2.0 Cell Analysis Software on the CellInsight CX7 High Content Analysis System. Error bars = SD; n = 3. Significance levels are indicated as follows: * *p* ≤ 0.05, *** *p* ≤ 0.001, **** *p* ≤ 0.0001.

Using the Human Protein Atlas (HPA) algorithm, we analyzed mRNA expression data of 362 liver cancer patients from the TCGA HCC cohort, and demonstrated that high TWF1 expression is associated with lower survival rates and a poor prognosis (Figure 6A). To further explore the role of TWF1 in HCC, we used specific TWF1 siRNAs (siTWF1 #5 and siTWF1 #6) to transiently knockdown TWF1 in TKI-resistant cells and observed a significant inhibition of proliferation and colony formation ability in all cell lines with TWF1 reduction (Figure 6B,C/Figure S6C,D). A cell cycle analysis demonstrated that silencing TWF1 with siRNA (siTWF1 #5) halted cell cycle progression at the G1/S phase in SNU475 and Huh-7/SR1 cells, while it led to a block at the G2/M phase in Huh-7/LR and PLC/PRF/5 cells (Figure 6D/Figure S6E). This finding was consistent with a reduction in Ki-67 staining in SNU475 and PLC/PRF/5 cells (Figure 6E/Figure S6F). Furthermore, siTWF1 also induced apoptosis in HCC cells (Figure 6F/Figure S6G), accompanied by an increased c-Casp3 positive area (Figure 6G,H/Figure S6H) and a cleaved PARP protein expression (Figure 6I). Consistent with the effects of miR-142-3p and siYES1, we observed that TWF1 depletion resulted in an increase in p21 expression (Figure 6I–K) and a decrease in CDC25C expression (Figure 6J,K), indicating the induction of cell cycle arrest and apoptosis. TWF1 knockdown significantly diminished the invasive capacity of SNU475 and Huh-7/LR cells in the Matrigel wound healing assay (Figure 6L,M), and impaired the chemotactic migration of Huh-7/LR cells in a 2D Transwell assay (Figure 6N). Additionally, TWF1 knockdown reduced the migratory potential of PLC/PRF/5 cells (Figure S6I). These findings indicate that TWF1 plays a crucial role in the proliferation, survival, migration, and invasion of HCC cells, and its association with a poor disease outcome in liver cancer patients highlights its potential as a therapeutic target in HCC.

### 2.7. MicroRNA-142-3p Restores Lenvatinib Sensitivity in TKI-Resistant HCC Cells Partially via Targeting YES1 and TWF1

Given the elevated levels of miR-142-3p in HCC patients responding to sorafenib, we investigated its role in restoring drug sensitivity in TKI-resistant HCC cell lines. To quantitatively evaluate the combinational effect of miR-142-3p and lenvatinib, we transiently transfected SNU475 and Huh-7/LR cells with an miR-142-3p mimic (or miR-NC) (0.01–100 nM) in a 3-fold dilution and, at 48 h post-transfection, the cells were treated with nine different concentrations of lenvatinib (0.1 to 50 µM). After 72 h of lenvatinib treatment, cell viability was assessed and the combination index (CI) of these two compounds was calculated using the Chou–Talalay method with CompuSyn software v. 1, where the CI values reflect the following: synergism (CI < 1), additive effect (CI = 1), or antagonism (CI > 1) [39].

**Figure 7:**
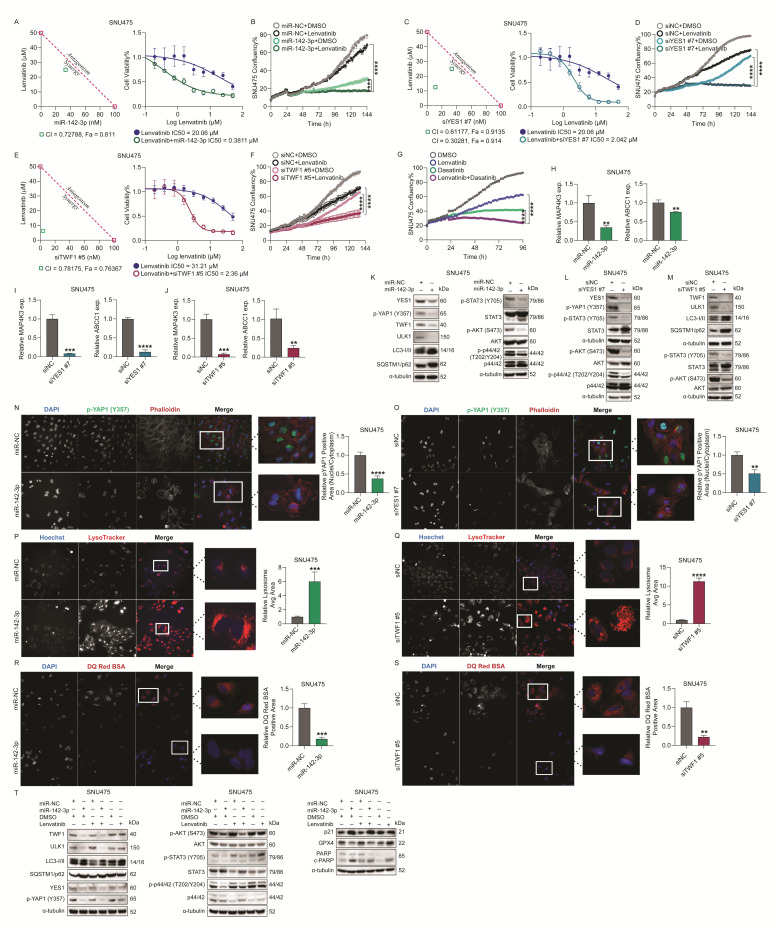
**Role of miR-142-3p and its gene targets in modulating TKI sensitivity in HCC cells.** (**A**) SNU475 cells were reverse-transfected with 10 different doses of miR-142-3p mimic or miR-NC in 1/3 serial dilutions (highest dose 100nM), and at 48 h post-transfection were treated with lenvatinib with 10 doses in 1/2 dilution (highest dose 50µM). After 72 h treatment, cell viability was determined using cell titer assay. The combination index (CI) value was determined using the Chou–Talalay method. The IC50 values of lenvatinib alone and in combination with miR-142-3p are shown in graphs. (**B**) Incucyte proliferation assay of miR-142-3p ± lenvatinib. SNU475 cells were transfected with either 5 nM miR-142-3p or miR-NC, and at 48 h post-transfection were treated with 10 µM lenvatinib or DMSO (vehicle control). Time-lapse imaging was conducted at 2-h intervals continuously for up to 144 h. (**C**–**F**) Similar experiments demonstrating the effects of siYES1 or siTWF1 in combination with lenvatinib, as described for miR-142-3p (**A,B**). The number following the “#” symbol refers to a specific siRNA targeting a defined sequence of the mRNA transcript, as designated by the manufacturer. (**G**) The Incucyte proliferation assay to assess the growth inhibition efficacy of the combination therapy with dasatinib at IC25 dose (14 µM) and lenvatinib at IC50 dose (12.8 µM) in SNU475 cells. (**H**–**J**) RT-qPCR analysis of MAP4K3 and ABCC1 mRNA expression in SNU475 cells transfected with a 10nM miR-142-3p (**H**), siYES1 #7 (**I**), or siTWF1 #5 (**J**) (or relevant negative controls) at 24 h post-transfection; data were analyzed using the 2^-ΔΔCt^ method with GAPDH as the reference gene. (**K**–**M**) SNU475 cells were transiently transfected with 10 nM miR-142-3p, siYES1 #7, or siTWF1 #5 or relevant negative controls and western blot analysis performed on lysates at 96 h post-transfection. α-tubulin was used as an internal control (housekeeping protein). (**N**,**O**) Immunofluorescence staining of p-YAP1 (Y357) to investigate its nuclear translocation in SNU475 cells transfected with 10nM miR-142-3p (**N**) or siYES1 #7 (**O**) for 96 h compared to the controls. (**P**,**Q**) Live cell imaging of SNU475 cells transfected with either a 10 nM miR-142-3p mimic (**P**) or siTWF1 #5 (**Q**) (or relevant controls) for 96 h, following labeling of lysosomes using 60 nM LysoTracker Deep Red. (**R**,**S**) SNU475 cells stained by DQ Red BSA at 96 h post-transfection with either 10 nM miR-142-3p mimic (**R**), siTWF1 #5 (**S**), or respective controls to measure lysosomal degradation activity. (**T**) Pathway analysis in SNU475 cells following combination of miR-142-3p mimic transfection (5nM) and lenvatinib (10 µM) treatment. After 48 h of transfection, the media was replaced with either lenvatinib- or DMSO-containing media and incubated for an additional 48 h. At 96 h post-transfection, cell lysates were collected for analysis. α-tubulin was used as an internal control (housekeeping protein). All immunofluorescence was using 20× magnification and the image analysis was performed using Thermo Fisher Scientific HCS Studio 2.0 Cell Analysis Software on the CellInsight CX7 High Content Analysis System. Error bars = SEM (**B**,**D**,**F**,**G**) or SD (**H**–**J**, **N**–**S**); n = 3. Significance levels are indicated as follows: ** *p* ≤ 0.01, *** *p* ≤ 0.001, **** *p* ≤ 0.0001.

Our measurements showed that, at several ratios, the CI values were less than 1 (Figure 7A), indicating a synergistic effect between miR-142-3p and lenvatinib. The shift in the lenvatinib IC50 value demonstrated how the concentration of miR-142-3p could improve the potency of lenvatinib (Figure 7A). This synergy was further validated using Incucyte proliferation assays in SNU475 cells (Figure 7B), Huh-7/LR, and PLC/PRF/5 cells (Figure S7A). Additionally, we investigated whether a combination of miR-142-3p (5nM) with sorafenib (10 µM) could restore sorafenib sensitivity of Huh-7/SR1 cells (Figure S7B), and found that the combination was synergistic; miR-142-3p effectively enhanced the sorafenib-mediated growth inhibition.

We further explored if downregulating the miR-142-3p targets YES1 and TWF1 contributes to restoring TKI sensitivity. The siRNA-mediated knockdown of either YES1 or TWF1 significantly reduced the IC50 of lenvatinib, indicating synergy (CI values below 1; Figure 7C, E). The Incucyte proliferation assay further validated synergy between lenvatinib and siYES1 #7 (Figure 7D/Figure S7G) or siTWF1 #5 (Figure 7F/Figure S7J) in multiple TKI-resistant HCC cell line models. Moreover, combining siYES1 #7 or siTWF1 #5 with sorafenib significantly enhanced the cytotoxic response in Huh-7/SR1 cells, demonstrating restored sensitivity to sorafenib (Figure S7H,K).

To additionally substantiate the effect of siYES1 mediated inhibition of HCC proliferation, we used dasatinib, a known YES1 inhibitor, and assessed its effect in combination with lenvatinib on HCC cell viability. This combination therapy markedly decreased cell viability in both SNU475 and Huh-7/LR cell lines more than either treatment alone, mirroring the synergistic effects observed with YES1 knockdown, and emphasizing the critical role of YES1 inhibition in boosting the efficacy of lenvatinib (Figure 7G/Figure S7I).

To further elucidate the role of miR-142-3p and its target genes in drug resistance, we transiently transfected miR-142-3p, siYES1 #7, or siTWF1 #5 (or relevant controls) into multiple HCC cell lines and analyzed the expression of drug resistance-associated genes MAP4K3 and ABCC1. The mRNA levels of MAP4K3 and ABCC1 were significantly decreased following miR-142-3p overexpression or knockdown of YES1 or TWF1 (Figure 7H–J and Figure S7C–E,L,M). Furthermore, western blotting confirmed the miR-142-3p targeting of key signaling pathways, including autophagy, STAT3, PI3K/AKT, YAP1, and p44/42 MAPK (Figure 7K/Figure S7F). Autophagy was evaluated by analyzing the protein expression levels of ULK1 (Unc-51-like kinase 1) and SQSTM1/p62, with miR-142-3p treatment resulting in decreased ULK1 expression and an accumulation of SQSTM1/p62, indicating disrupted autophagy (Figure 7K and Figure S7F). Furthermore, the expression levels of LC3-II, a protein essential for autophagosome maturation and fusion with lysosomes [37], were also increased following miR-142-3p overexpression (Figure 7K), suggesting either enhanced autophagosome formation or a blockade in autophagic flux.

Knockdown of YES1 in SNU475 cells recapitulated the effects of miR-142-3p on the regulation of STAT3, PI3K/AKT, YAP1, and MAPK pathways (Figure 7L). YES1 phosphorylates YAP1 is a transcriptional co-activator of the Hippo signaling pathway, at tyrosine 357, and promotes its nuclear translocation, thereby enhancing the expression of genes involved in cell survival and proliferation, ultimately contributing to the development of chemoresistance [40]. Using immunofluorescence staining of phospho-YAP1 (Y357) followed by automated high-content imaging and image analysis, we demonstrated that both the miR-142-3p mimic and the siYES1 #7 treatment of SNU475 cells inhibited YAP1 phosphorylation and subsequent nuclear translocation in HCC cells (Figure 7N,O).

TWF1 knockdown modulated the STAT3 and PI3K/AKT pathways and autophagy, as evidenced by changes in ULK1 and SQSTM1/p62 levels, and increased LC3-II protein (Figure 7M). These findings were further supported by the increased LysoTracker intensity observed in SNU475 cells overexpressing miR-142-3p, and in cells with TWF1 knockdown, compared to their respective controls (Figure 7P,Q). This increased intensity indicates either a larger number of lysosomes or an increase in lysosomal size, which is commonly associated with autophagy inhibition [41]. This finding correlates with our RNA-seq data, further highlighting the downregulation of genes involved in lysosomal function, organelle membrane fusion, and autophagosome organization (Figure 4B,C, Table S5/Table S6). Moreover, an RT-qPCR analysis of miR-142-3p overexpressing SNU475 and Huh-7/LR cells showed a significant reduction in *STX12* and *SLC17A5* gene expression (Figure S7N,O). *STX12* is crucial for the fusion of autophagosomes with lysosomes, facilitating the degradation of cellular components, while *SLC17A5* is responsible for the transport of sialic acid and related metabolites across lysosomal membranes [42,43]. The downregulation of these genes suggests that miR-142-3p may impair autophagic flux and lysosomal function, potentially contributing to altered cellular homeostasis. To further validate our observations, we performed immunofluorescence staining using DQ Red BSA to assess lysosomal activity. The results demonstrated a significant reduction in BSA degradation in SNU475 cells transfected with the miR-142-3p mimic or siTWF1 #5 compared to the controls, as evidenced by decreased fluorescence intensity (Figure 7R,S). Based on the experiments conducted, we can conclude that both miR-142-3p overexpression and TWF1 knockdown lead to a blockade of the autophagy process in HCC cells.

A pathway analysis of SNU475 cells treated with miR-142-3p (5 nM) and lenvatinib (10 µM) demonstrated suppression of YES1, TWF1, and their effectors, leading to decreased YAP1 activity and autophagy flux. This treatment also induced apoptosis and ferroptosis, evidenced by elevated cleaved PARP and p21 levels and reduced GPX4 (Figure 7T). Collectively, these findings highlight the potential of miR-142-3p or inhibitors of its downstream targets, YES1 and TWF1, to restore drug sensitivity in HCC cells.

## 3. Discussion

New treatment strategies for HCC are urgently needed, and specific tumor suppressor miRNAs have emerged as key modulators of HCC progression and therapy resistance. Here, using an in silico approach, we identified miR-142-3p as a miRNA with therapeutic potential in HCC, with its overexpression resulting in a marked reduction in HCC cell line proliferation, colony formation, migration/invasion, as well as decreased expression of EMT-associated genes (e.g., *VIM*, *CDH2*, *SNAI2*, *ZEB1*, and *ZEB2*), and an increase in cell cycle arrest and apoptosis. Previous studies have identified miR-142-3p to be a profound tumor suppressor in various cancer types, including melanoma [44], colorectal [45], breast [46], ovarian [47], cervical [48], lung [49], and HCC [50], highlighting its critical role in cancer biology and therapeutic outcomes. In HCC, miR-142-3p has multiple specific functions, including promoting ferroptosis, inhibiting metastasis, sensitizing cells to sorafenib, and modulating key oncogenes (e.g., high-mobility group box 1 [51]) and metabolic enzymes (e.g., lactate dehydrogenase A [50]) [51,52,53,54]; however, the precise mechanisms by which miR-142-3p modulates liver cancer progression and therapeutic resistance remains to be elucidated. Herein, we identified *YES1* and *TWF1* as direct target genes of miR-142-3p. Specific targeting of these genes reduced HCC cell survival and invasiveness, and therefore holds therapeutic potential for HCC. Furthermore, an miR-142-3p mimic or silencing YES1 and TWF1 with siRNAs can restore lenvatinib sensitivity in TKI-resistant cells.

In recent years, inhibitors targeting key signaling pathways, such as AKT, MAPK/ERK, and STAT3, have gained prominence in cancer therapy due to their critical roles in the regulation of tumor growth, survival, and metastasis, thereby making them prime targets for therapeutic intervention [55]. AKT inhibitors in clinical trials include ipatasertib (Phase III trials, NCT04650581) [56] and capivasertib, an ATP-competitive pan-Akt inhibitor (in Phase II/III trials [57]), both of which have shown promising results in breast cancer. Similarly, MAPK inhibitors e.g., ulixertinib (Phase II trials [58]) and STAT3 inhibitors, such as TTI-101 (OPB-111077) (phase I clinical trial (NCT03195699) [59]), have demonstrated antitumor activity in patients with advanced stage solid tumors.

Sorafenib, a well-established inhibitor of the RAF/MAPK/ERK pathway, is known to impede angiogenesis and promote apoptosis in HCC [60]. However, these inhibitors targeting specific pathways often encounter resistance issues, as cancer cells can activate compensatory signaling mechanisms that allow them to survive and proliferate despite ongoing treatment, highlighting the necessity for more comprehensive treatment strategies capable of simultaneously disrupting several signaling pathways [55]. Our findings reveal that miR-142-3p can simultaneously disrupt the AKT, MAPK/ERK, and STAT3 signaling pathways, which has significant clinical implications for overcoming therapeutic resistance. The SRC family kinases (SFKs) are non-receptor tyrosine kinases identified as proto-oncogenic drivers. YES1 is an SFK and is frequently amplified and overexpressed in various human malignancies, including lung, breast, ovarian, and skin cancers [32], and has been increasingly recognized for its role in promoting cell proliferation, survival, invasiveness, and chemoresistance [31,61]. Combining YES1 inhibitors with taxanes has been shown to improve treatment efficacy by inducing maladaptive chromosomal instability, thereby reducing the survival of triple-negative breast cancer cells [62]. Dasatinib, an FDA-approved and potent multitargeted tyrosine kinase inhibitor, targets several SFKs, including YES1 [63]. In colorectal cancer, dasatinib has demonstrated efficacy in restoring oxaliplatin sensitivity in tumors characterized by elevated p-Src levels [64]. Additionally, preclinical studies have highlighted the potential of dasatinib in combination with anti-PD-1 antibody therapy for the treatment of metastatic colorectal cancer [65]. Our experimental data shows that the combination of YES1 inhibition (via siRNAs or dasatinib) with lenvatinib yields stronger antiproliferative effects than lenvatinib alone in HCC cells, which supports the notion of combination therapy as a promising strategy to overcome TKI resistance in the treatment of HCC in clinical settings.

Furthermore, the broader significance of SFK inhibition extends to its interaction with the Hippo signaling pathway, where it can prevent the stabilization and nuclear localization of YAP1, a key modulator of tumor progression. YES1 is implicated in YAP1’s activation. Research by Guégan et al. highlighted YAP and TAZ as essential mediators of YES1-induced transformation in hepatocytes, noting that increased YES1 activity is associated with poor prognosis in some HCC cases [66]. Small molecule inhibitors targeting the YAP/TAZ-TEAD transcription factor complexes have shown promising preclinical efficacy in mouse models of mesothelioma; these are undergoing clinical evaluation in trials, e.g., ION537 (NCT04659096), VT3989 (NCT04665206), IK-930 (NCT05228015), and IAG933 (NCT04857372) [67]. Considering these findings, our results demonstrate that using miR-142-3p or siYES1 presents a promising approach to inhibit YAP1 activation and its nuclear localization, offering a potential strategy to disrupt tumor growth.

TWF1 is overexpressed in multiple cancers, including lung adenocarcinoma, breast cancer, and pancreatic cancer, and predicts poor outcomes [25]. In pancreatic cancer, TWF1 downregulation by miR-142-3p reduces cell proliferation and EMT, while enhancing apoptosis in gemcitabine-resistant pancreatic cancer cells [38]. In lung adenocarcinoma, downregulation of TWF1 suppresses invasion, migration, and autophagy [37]. Our research builds on these observations by identifying TWF1 as a direct target of miR-142-3p in HCC cells, demonstrating that both overexpression of miR-142-3p and knockdown of TWF1 stimulated lysosomal malfunction and inhibited autophagy, thereby restoring TKI sensitivity in HCC cell lines. This lysosomal malfunction prevents the critical degradation steps within autophagolysosomes, resulting in profound changes in lysosomal dynamics and capacity, significantly impacting cellular homeostasis and survival. Further corroborating our findings, Zhang et al. reported that miR-142-3p increases the susceptibility of HCC cells to sorafenib by downregulating autophagy-promoting genes ATG5 and ATG16L1, enhancing apoptosis and reducing cell proliferation [54]. Moreover, miR-142-3p is implicated in regulating autophagy in non-small cell lung cancer (NSCLC), thereby enhancing the sensitivity of NSCLC cells to cisplatin [68]. In colorectal cancer, upregulation of miR-142-3p augments the sensitivity of cancer cells to 5-fluorouracil by targeting autophagy-related proteins [69]. Shi et al. [70] reported a reduction of miR-142-3p expression in paclitaxel (PTX)-resistant breast cancer cell lines compared to parental cells. Overexpression of miR-142-3p diminished the viability and migration of these drug-resistant cells and disrupted autophagic flux, while simultaneously enhancing apoptosis and PTX sensitivity [70]. These observations highlight TWF1 and miR-142-3p as pivotal factors in cancer biology, suggesting that their modulation could be crucial steps in developing more effective treatment strategies for therapy-resistant cancers.

Inhibiting autophagy and lysosomal function is emerging as a promising strategy in cancer treatment, especially considering that many cancer cells rely on these processes for survival under therapeutic stress. Autophagy allows tumor cells to recycle cellular components, thus supporting their growth during nutrient deprivation, while lysosomes play a critical role in the degradation of autophagosomes necessary for maintaining cellular homeostasis [71]. Hydroxychloroquine (HCQ) and chloroquine (CQ) are antimalarial drugs that inhibit autophagy by disrupting lysosomal acidification and are currently being tested in combination with various cancer therapies in a phase II trial [72]. Additionally, SAR405 is an innovative ATP-competitive inhibitor of vacuolar protein sorting 34 (VPS34), a class III phosphoinositide 3-kinase (PI3K) that plays a crucial role in autophagy, endocytosis, and membrane trafficking. By specifically targeting VPS34, it disrupts autophagy and impedes the trafficking of late endosomes to lysosomes, demonstrating preclinical efficacy in tumor types that rely heavily on autophagic processes [73]. Lastly, MRT67307 is an ULK1 inhibitor that has demonstrated potential anti-tumor activity by impairing autophagy [74]. Our findings reveal that miR-142-3p overexpression not only reduces ULK1 protein levels but downregulates lysosomal function-associated gene SLC17A5, and the autophagosome–lysosome attachment gene STX12, highlighting its potential to disrupt autophagy and lysosomal processes in cancer cells. ABCC1, a member of the ATP-binding cassette (ABC) transporter family, plays a key function in mediating chemoresistance by facilitating the efflux of chemotherapeutic drugs from cancer cells, thereby diminishing drug effectiveness [75]. Elevated ABCC1 levels in HCC are associated with an unfavorable prognosis, suggesting that ABCC1 is a potential indicator of treatment response and a viable target for enhancing therapeutic outcomes [75]. Further, MAP4K3 regulates mTORC1 signaling, impacting cell survival [76], and contributes to drug resistance by helping cancer cells adapt to metabolic stress [77]. In this study, we report that overexpression of miR-142-3p or knockdown of YES1 and TWF1 notably reduced the mRNA expression of ABCC1 and MAP4K3 in HCC cells, further illustrating the role of these three molecules as regulators of chemoresistance in these cellular models. While our study primarily focused on tumor cell–intrinsic mechanisms of lenvatinib resistance, it is important to acknowledge that in vivo resistance can also arise from adaptations within the tumor microenvironment. Specifically, the activation of alternative angiogenic pathways—such as those involving angiopoietin-2 (Ang-2) [78,79], hepatocyte growth factor (HGF/c-Met) [80,81], and interleukin-8 (IL-8) [82,83]—has been shown to restore neovascularization and tumor perfusion independently of VEGF/FGF signaling. These non-VEGF escape mechanisms represent well-documented contributors to anti-angiogenic therapy resistance. However, evaluating the influence of miR-142-3p on these TME-mediated pathways was beyond the scope of this study, which was designed to dissect direct tumor cell-based mechanisms.

In recent years, advancements in RNA-based therapies have significantly transformed the landscape of medical treatments, offering innovative solutions for previously challenging diseases. Examples of clinical successes include the COVID-19 mRNA vaccines [84] and siRNA-based therapeutics, such as *patisiran* and *inclisiran*, offering targeted treatments for hereditary transthyretin amyloidosis and hypercholesterolemia, respectively [84]. These advancements have been supported by modifications in RNA chemistry, which enhance the stability and efficacy in vivo, along with improvements in targeted delivery methods, such as lipid nanoparticles (LNPs), which facilitate efficient delivery to specific cells or tissues [84]. Our future studies will aim to incorporate some of these advancements in RNA chemistry and delivery methods to evaluate the potential of miR-142-3p as an RNA-based therapeutic in animal models, thereby enhancing its translational potential and therapeutic applicability.

## 4. Materials and Methods

### 4.1. Chemicals and Reagents

A complete list of chemicals, miRNAs, siRNAs, antibodies, and primers used in this study, together with specific catalogue numbers and companies is provided in Supplementary Table S2.

### 4.2. Data Retrieval from Publicly Available Databases

The Cancer Cell Line Encyclopedia (https://www.broadinstitute.org/ccle/) and the Cancer Therapeutics Response Portal (CTRP) (https://www.broadinstitute.org/scientific-community/software/cancer-therapeutics-response-portal) were used to extract miRNA expression profiles and drug sensitivity data for TKIs, including sorafenib, lenvatinib, and cabozantinib, from 791 human cancer cell lines. Basal miRNA levels were ranked based on the response of each cell line to TKIs by evaluating the correlation coefficients as previously described [85]. Clinical data was also extracted from an HCC cohort (the STORM TRIAL, BIOSTORM cohort) [86], sourced from the GSE109211 dataset on the Gene Expression Omnibus (GEO) database (https://www.ncbi.nlm.nih.gov/geo/). The Kaplan–Meier Plotter tool (https://kmplot.com/analysis/) was used to generate the overall survival (OS) plot for miR-142-3p using the non-commercial spotted platform (n = 166). Expression thresholds were set at cutoff-high and cutoff-low to divide the cohorts into high-expression and low-expression groups. Additionally, the correlation between the *TWF1* mRNA expression levels and patient survival was examined using data from The Human Protein Atlas (HPA) database (https://www.proteinatlas.org/).

### 4.3. Cell Culture

SNU475, SNU423, and PLC/PRF/5 cell lines were sourced from the American Type Culture Collection (ATCC, Manassas, VA, USA). Huh-7 cells were gifted by Prof. Nicholas Shackel, Ingham Institute at Liverpool Hospital, Sydney, Australia. Acquired sorafenib-resistant Huh-7/SR1 and lenvatinib-resistant Huh-7/LR cell lines were developed in-house as previously described [87], by maintaining the cells in gradually increasing concentrations of TKIs. All cell lines were subjected to short tandem repeat (STR) typing to validate their origin. SNU475 and SNU423 cells were cultured in RPMI 1640 medium (Gibco, Thermo Fisher Scientific, Waltham, MA, USA; Cat# 11875093) supplemented with 10% heat-inactivated fetal bovine serum (FBS; Sigma-Aldrich, St. Louis, MO, USA; Cat# F9423). PLC/PRF/5 cells were cultured in minimum essential medium (MEM; Gibco, Thermo Fisher Scientific, Waltham, MA, USA; Cat# 11095080) supplemented with 10% FBS, 1 mM sodium pyruvate (Gibco, Thermo Fisher Scientific, Waltham, MA, USA; Cat# 11360070), and non-essential amino acid solution (NEAA; Gibco, Thermo Fisher Scientific, Waltham, MA, USA; Cat# 11140050). Huh-7, Huh-7/SR1, and Huh-7/LR cells were cultured in low-glucose Dulbecco’s modified Eagle medium (DMEM; Gibco, Thermo Fisher Scientific, Waltham, MA, USA; Cat# 11885084) supplemented with 10% FBS. Cells were maintained in an incubator at 37 °C and 5% CO_2_, and were routinely monitored for mycoplasma contamination.

### 4.4. Transfection of miRNA and siRNA Molecules

Cells were transiently transfected with the miR-142-3p mimic/inhibitor, siRNAs, or corresponding negative controls (miR-NC and siNC) at a concentration of 1–10 nM, using Lipofectamine 2000 according to the manufacturer’s instructions. Seeding densities and the choice of plates varied according to the requirements of each specific assay, details of which are specified in the sections below.

### 4.5. RNA Isolation and Real-Time-Quantitative Polymerase Chain Reaction (RT-qPCR) Assay

Total RNA was isolated 24 h post-transfection using TRIzol Reagent (Thermo Fisher Scientific, Waltham, MA, USA) as per the manufacturer’s instructions. Total RNA (800 ng) was reverse transcribed into cDNA using the QuantiTect Reverse Transcription Kit (Qiagen, Hilden, Germany). Subsequent PCR was performed on a Qiagen Rotor-Gene Q real-time PCR system using SensiMix SYBR Hi-ROX (Bioline, London, UK) and gene specific primers (see Table S2). For quantification of mature miRNAs, the TaqMan MicroRNA Reverse Transcription Kit and the TaqMan MicroRNA Assay (Applied Biosystems, Foster City, CA, USA) were used. The relative abundance of each target gene was normalized to the expression levels of housekeeping genes (GAPDH or HPRT) or a small RNA (U6 snRNA), and the fold change was calculated in comparison to the negative control using the 2^−ΔΔCt^ method.

### 4.6. Protein Isolation and Western Immunoblotting

To analyze protein expression, cells were lysed 96 h post-transfection to extract whole cell lysate using a cell lysis buffer (Cell Signaling Technology, Danvers, MA, USA) supplemented with the phosphatase inhibitor phosSTOP (Roche, Basel, Switzerland), protease inhibitor cocktail (Roche, Basel, Switzerland), and 1 mM PMSF (Sigma-Aldrich, St. Louis, MO, USA). Total protein concentration was determined using the Bradford assay (Bio-Rad Laboratories, Hercules, CA, USA). Subsequently, proteins (30–50 µg) were resolved on NUPAGE 4–12% Bis-Tris gels (Thermo Fisher Scientific, Waltham, MA, USA) and transferred to polyvinylidene difluoride (PVDF) membranes (Millipore, Burlington, MA, USA). For immunodetection of specific proteins, membranes were blocked with 5% skim milk/Tris-buffered saline Tween 20 (TBS-T) for 1 h, and then incubated with primary antibodies diluted in 5% bovine serum albumin (BSA)/TBS-T overnight at 4 °C. The following day, the membranes were washed with blocking buffer and incubated with horseradish peroxidase (HRP)-conjugated secondary antibody for 1 h at room temperature. After additional washes in TBS-T, the membranes were incubated with Immobilon Crescendo Western HRP substrate (Merck, Darmstadt, Germany), and protein bands were visualized using the iBright Imaging System (Thermo Fisher Scientific, Waltham, MA, USA). Detailed information on the antibodies used is provided in Table S2.

### 4.7. Incucyte Proliferation Assay

For the cell proliferation assay, 3.5–5 × 10^3^ cells/well were reverse transfected with miRNA mimics/inhibitors or siRNAs (or their respective controls: miR-NC, anti-miR-NC, and siNC) and seeded into a 96-well plate at a final concentration ranging from 1–10 nM per well. The plates were incubated in an Incucyte Zoom automated live cell imaging system (Sartorius) in a 37 °C humidified incubator with 5% CO_2_, and cell growth was continuously monitored by imaging at two-hour intervals for a period ranging from 72 to 144 h. An image analysis via the built-in Incucyte Zoom software (Sartorius, Göttingen, Germany) was employed to measure cell confluence (%).

### 4.8. Cell Cycle and Annexin V-FITC/PI Apoptosis Assays

For cell cycle and Annexin V fluorescein isothiocyanate (FITC)/propidium iodide (PI) apoptosis assays, HCC cells were plated into six-well plates (25 × 10^4^ cells/well), transfected with 10 nM miRNA mimic, siRNA, or their respective negative controls, and cells were harvested at 72 h post-transfection. For cell cycle analysis, 1 × 10^6^ cells were harvested, fixed in cold 70% ethanol, treated with RNase A (5 µg/mL) at 37 °C for 30 min, and stained with PI (50 µg/mL). Subsequently, cells were analyzed for their cell cycle distribution using an Accuri C6 flow cytometer (BD Biosciences, San Jose, CA, USA) and FlowJo 7.6 data analysis software (BD Biosciences, San Jose, CA, USA). An apoptosis analysis was performed using the Annexin V-FITC Apoptosis Detection Kit (BD Biosciences, San Jose, CA, USA) and Accuri C6 flow cytometer, following the manufacturer’s protocol. Gating using unstained or single-stain cells was applied to differentiate between live, apoptotic, and dead cell populations, and the data were analyzed using BD Accuri C6 Plus software, version 1.0.264 (BD Biosciences, San Jose, CA, USA).

### 4.9. Incucyte Wound Healing Assay

Cell migration and invasion were assessed using a wound healing assay performed in Incucyte Imagelock 96-well plates (Sartorius, Göttingen, Germany), with and without Matrigel, respectively. HCC cells were initially reverse-transfected in 10-cm culture dishes with 10 nM miRNA mimics, siRNAs, or their respective negative controls. After 24 h, the transfected cells were trypsinized, counted, and seeded at a density of 1 × 10^5^ cells per well in 96-well plates, with six replicates for each condition. The plates were incubated overnight to allow the cells to form a confluent monolayer. On the following day, to eliminate the impact of proliferation and to synchronize the cells, mitomycin C (Sigma-Aldrich, St. Louis, MO, USA) was applied at a concentration of 2 µg/mL for 15 min at 37 °C. After treatment, mitomycin C was removed and 100 µL of complete medium was added to each well. A uniform scratch was then made in each well using the Woundmaker tool (Essen BioScience, Ann Arbor, MI, USA), followed by an additional wash step to remove detached cells and debris. For the migration assay, 200 µL of complete growth medium was added to each well immediately after the scratch. For the invasion assay, 50 µL of Matrigel (Corning, Corning, NY, USA) diluted to a final concentration of 1 mg/mL was added directly on top of the scratched monolayer immediately following the scratch. The plate was incubated at 37 °C for 30 min to allow the Matrigel to solidify, after which 150 µL of complete medium was added to each well.

Cell migration and invasion into the wound area were monitored in real time using the Incucyte Zoom live-cell imaging system (Sartorius, Göttingen, Germany). An image analysis was performed using the Incucyte Scratch Wound Analysis software, version 2018A (Essen BioScience, Ann Arbor, MI, USA) to calculate wound confluence (%) and relative wound density (%), which reflect the area covered by migrating or invading cells over time.

### 4.10. Transwell Chemotaxis Assay

Cell motility was further assessed using a transwell chemotaxis assay in 24-well plates with polycarbonate membrane inserts with an 8.0 µm pore size (Costar, Corning, NY, USA). Cells (1 × 10^5^) were seeded into the upper chamber of the plates with 400 μL of DMEM containing 1% FBS, and the lower chamber was filled with 500 μL of DMEM supplemented with 10% FBS. After incubation at 37 °C for 48 h, inserts were removed from the wells. The non-migrated cells on the upper surface were gently scraped off using a cotton swab and the membranes were fixed with methanol (Chem-Supply, Gillman, SA, Australia) for 10 min and subsequently stained with 0.1% crystal violet (Sigma-Aldrich, St. Louis, MO, USA) for 5 min. The migrated cells were counted under an Olympus IX71 Inverted Fluorescence Microscope (Olympus Corporation, Tokyo, Japan), and six images were captured per well at 20× magnification. ImageJ software, version 1.54i(National Institutes of Health, Bethesda, MD, USA) was employed to analyze and quantify the number of migrated cells.

### 4.11. Colony Formation Assay

For colony formation assays, 5 × 10^2^ pre-transfected cells per well were seeded in a six-well plate. Following a two-week incubation period, the colonies were fixed with cold 100% methanol (Chem-Supply, Gillman, SA, Australia) for 10 min and then stained with 0.1% crystal violet (Sigma-Aldrich, St. Louis, MO, USA) in methanol for 5 min. The plates were scanned using the iBright Imaging System (Thermo Fisher Scientific, Waltham, MA, USA), and colonies were counted using the ImageJ software, version 1.54i (National Institutes of Health, Bethesda, MD, USA). A colony was defined as a cluster consisting of at least 50 cells.

### 4.12. Immunofluorescence Assay

Cells were fixed at 96 h post-transfection, with 4% paraformaldehyde (Sigma-Aldrich, St. Louis, MO, USA) at room temperature for 10 min, washed three times with phosphate buffered saline (PBS), permeabilized with 0.1% Triton X-100 (Sigma-Aldrich, St. Louis, MO, USA) in PBS for 5 min, and blocked in 5% BSA (Sigma-Aldrich, St. Louis, MO, USA) in PBS for 1 h. Subsequently, primary antibodies—cleaved caspase 3 (c-Casp3, Cell Signaling Technology, Danvers, MA, USA; Cat# 9661; 1:500 dilution), phospho-YAP1 (p-YAP1) (Y357) (Abcam, Cambridge, UK; Cat# ab62751; 1:1000 dilution), and Ki67 (Abcam, Cambridge, UK; Cat# ab15580; 1:1000 dilution)—were prepared in 1% BSA/PBS, added to the cells, and incubated overnight at 4 °C. The following day, after washing twice with blocking buffer, the cells were incubated with Alexa 488-conjugated goat anti-rabbit IgG secondary antibody (Thermo Fisher Scientific, Waltham, MA, USA; Cat# A-11008; 1:1000) for 1 h at room temperature. Subsequently, the cells were counterstained with 4′,6-diamidino-2-phenylindole (DAPI) (Sigma-Aldrich, St. Louis, MO, USA; 1:1000 dilution) for nuclei/cell number detection, and Rhodamine Phalloidin (Biotium, Fremont, CA, USA; 1:500 dilution; Cat# 00027), which specifically binds to F-actin for cell morphology and cytoskeletal structure assessment for 30 min. Imaging of the plates was performed using the CellInsight CX7 high-content screening platform (Thermo Fisher Scientific, Waltham, MA, USA) utilizing three channels: blue (380 nm, for DAPI), green (488nm, for c-Casp3, p-YAP1 (Y357), or Ki67), and red (590 nm, for phalloidin). HCS Studio software, version 6.6.2 (Thermo Fisher Scientific, Waltham, MA, USA) was used for image analysis.

We identified regions stained for c-Casp3 (marker of apoptosis) or Ki67 (marker of proliferation) as discrete spots and measured the average intensity of these spots per well, along with the total area represented by c-Casp3 or Ki67 staining. To calculate the positive area stained for our targets, we used the following formula: Positive Area = Average Intensity × Total Area. To assess the translocation of p-YAP1 (Y357), we measured the average intensity of spots in both the nuclei (stained with DAPI) and the cytoplasm (stained with phalloidin). We then calculated the ratio of the p-YAP1 (Y357) average intensity in the nuclei to that in the cytoplasm.

### 4.13. LysoTracker Deep Red and DQ Red BSA Staining

LysoTracker Deep Red and DQ Red BSA were used to visualize lysosomes and lysosomal activity, respectively. SNU475 cells were reverse transfected with 10 nM miRNA mimic, siRNA, or their respective negative controls in 96-well plates and incubated for 96 h. For lysosome staining, 60 nM LysoTracker Deep Red (Thermo Fisher Scientific, Waltham, MA, USA), together with 1 μg/mL Hoechst 33,342 (Sigma-Aldrich, St. Louis, MO, USA) for nuclei visualization, were added to the cells in complete culture medium and incubated for an additional 30 min in a humidified incubator. Subsequently, the dye was removed, and the cells were maintained in complete culture medium to conduct live cell imaging.

For lysosomal activity assessment, 100 μL of DQ–Red–BSA (Thermo Fisher Scientific, Waltham, MA, USA) at 10 μg/mL in MEM medium supplemented with 1% fetal bovine serum (FBS; Sigma-Aldrich, St. Louis, MO, USA; Cat# F9423), 1% non-essential amino acids (NEAA; Gibco, Waltham, MA, USA; Cat# 11140050), 1% GlutaMAX (Gibco, Waltham, MA, USA; Cat# 35050061), and 1% HEPES (Gibco, Waltham, MA, USA; Cat# 15630130), was added to the cells and incubated for 1.5 h at 37 °C with 5% CO_2_. The dye was then removed, and the cells were fixed with 4% PFA (Sigma-Aldrich, St. Louis, MO, USA) in PBS, followed by DAPI (Sigma-Aldrich, St. Louis, MO, USA) staining for 30 min. Then, the cells were washed with, and maintained in, PBS before imaging. For both assays, the plates were scanned at 20× magnification using the CellInsight CX7 high-content screening platform (Thermo Fisher Scientific, Waltham, MA, USA) using the blue channel (380 nm) for Hoechst and DAPI, the red channels (590 nm or 650 nm) for DQ–Red–BSA and LysoTracker, and image analysis was conducted using HCS Studio software, version 6.6.2 (Thermo Fisher Scientific, Waltham, MA, USA). The lysosomal average area within the cells was quantified using the following formula: Lysosome Average Area = Average Intensity × Total Area. Similarly, the quantification of the DQ–Red–BSA positive area was calculated as the product of average intensity and total area.

### 4.14. RNA Sequencing and Bioinformatic Analysis

Total RNA was extracted from SNU475 cells transfected with 10 nM miR-NC or miR-142-3p mimic for 24 h (four replicates per group) using a RNeasy Mini Kit (Qiagen, Hilden, Germany; Cat# 74104) according to the manufacturer’s instructions. RNA quantity and purity were evaluated using an RNA 6000 Nano Kit and Bioanalyzer 2100 (Agilent Technologies, Santa Clara, CA, USA) and a Qubit RNA IQ Assay (Thermo Fisher Scientific, Waltham, MA, USA), respectively. Stranded PolyA RNA-seq libraries were prepared and sequenced by the Genomics WA Core Facility, University of Western Australia (Perth, WA, Australia).

RNA sequencing data were processed using the Genomics WA AGeNT HS2 RNA-seq pipeline, version 1.51. Raw data was demultiplexed using bcl2fastq, version 2.20 (Illumina, San Diego, CA, USA) and quality control of FASTQ reads was performed using FastQC, version 0.11.9 (Babraham Bioinformatics, Cambridge, UK). Following initial quality checks, adapter sequences were trimmed using AGeNT Trimmer, version 2.0.3 (Agilent Technologies, Santa Clara, CA, USA). Paired-end reads were aligned using STAR, version 2.7.5a [88] to the human genome (hg38 build). Transcripts were quantified using featureCounts, version 2.0.1, and GENCODE Gene annotation (Human Release 35). Data normalization and differential expression analyses were conducted using the DESeq2 package, version 1.32.0 in R [89]. The *p*-values were calculated using the Wald test integrated in DESeq2, and these were then adjusted for multiple hypothesis testing using the Benjamini–Hochberg false discovery rate (FDR) method to derive adjusted *p*-values. Differentially expressed genes (DEGs) were identified by setting a threshold of 5% false discovery rate (FDR < 0.05) [90], and at least a two-fold change in expression between groups.

Gene ontology and pathway analyses were conducted using Gene Set Enrichment Analysis (GSEA) software, version 4.1.0 (Broad Institute, Cambridge, MA, USA) [http://www.broadinstitute.org/gsea/index.jsp] and Enrichr (Icahn School of Medicine at Mount Sinai, New York, NY, USA) [http://amp.pharm.mssm.edu/Enrichr/]. To generate enrichment plots using the GSEA tool, a Gene Ontology Biological Process (GOBP) analysis was performed using c5.go.bp.v2023.1.Hs.symbols.gmt gene set file, and Kyoto Encyclopedia of Genes and Genomes (KEGG) functional enrichment analysis was conducted using the c2.cp.kegg_legacy.v2023.2.Hs.symbols.gmt curated gene set database. A total of 1000 permutations were applied, with gene sets constrained between 15 and 500 genes. To evaluate enrichment levels and statistical significance, the software calculated normalized enrichment scores (NES) and FDR q-values. The acceptance criteria for functional pathways or processes were stringent, requiring an absolute NES value greater than 1, a nominal (NOM) *p*-value less than 0.05, and an FDR threshold < 25% to determine significant enrichment.

### 4.15. Luciferase Reporter Assay

The miRNA 3′-UTR luciferase reporter constructs for YES1 and TWF1 were generated by GeneCopoeia (Rockville, MD, USA) using the pEZX-MT06 vector. These constructs included both the wild-type miR-142-3p binding sites at positions 1588–1599 for TWF1 and 2404–2411 for YES1, as well as the corresponding mutated sequences (see Supplementary Materials: Table S2). Huh-7 cells were co-transfected in six-well plates with 25 nM miR-142-3p or miR-NC and 500 ng of the reporter plasmids using Lipofectamine 2000 (Thermo Fisher Scientific, Waltham, MA, USA). After 24 h, total protein was extracted from the transfected cells using a passive lysis buffer (Promega, Madison, WI, USA) and quantified using the Bradford assay (Bio-Rad Laboratories, Hercules, CA, USA). Equal amounts of protein (15 µg) were dispensed into each well of a white 96-well plate. Luciferase activity was measured using a Luciferase Reporter Assay System (Promega, Madison, WI, USA), as per the manufacturers’ instructions, using a CLARIOstar Plus microplate reader (BMG LABTECH, Ortenberg, Germany). The results were normalized to miR-NC control.

### 4.16. Dose–Response Curves, Determination of IC50 Values, and Combinational Synergy Assays

SNU475 and Huh-7/LR cells were seeded at a density of 5 × 10^3^ cells per well in 96-well plates. The following day, cells were treated with nine different concentrations of each drug (ranging from 0.01 to 50 µM) in a 2-fold dilution series, with six technical replicates. Cell viability was assessed at 72 h and 48 h post-treatment with lenvatinib (Selleck Chemicals, Houston, TX, USA; Cat# S5240) and dasatinib (Selleck Chemicals, Houston, TX, USA; Cat# S1021), respectively, using a CellTiter 96 AQueous One Solution Cell Proliferation Assay (Promega, Madison, WI, USA) according to the manufacturer’s guidelines. Absorbance was measured at 490 nm using a CLARIOstar Plus microplate reader (BMG LABTECH, Ortenberg, Germany). The half-maximal inhibitory concentration (IC50) values were calculated using GraphPad Prism, version 10.1.2 (GraphPad Software, San Diego, CA, USA), applying nonlinear regression with a four-parameter logistic equation (log (inhibitor) vs. response-variable slope) to generate dose–response curves. For synergy assays, the Chou–Talalay method [39] was used. SNU475 and Huh-7/LR cells were reverse-transfected with miRNA mimic or siRNA (0.01–100 nM) in a 3-fold dilution series using the above-described workflow. At 48 h post-transfection, the cells were treated with nine different concentrations of lenvatinib, ranging from 0.1 to 50 µM. After 72 h of lenvatinib treatment, cell viability was assessed and dose–response curves and IC50 values in the presence or absence of miRNA or siRNA were calculated as above. Additionally, the fraction affected (FA), was calculated using the following formula: FA = 1 − OD, where OD represents the optical density reflecting cell viability. The FA quantifies the proportion of cells affected by the treatment, with higher FA values indicating greater efficacy. This measure allows for the precise calculation of drug interactions by comparing the FA at different doses, thereby enabling a more accurate interpretation of the synergistic or antagonistic effects of the drug combinations. Isobolograms with ≥70% inhibition of cancer cell proliferation were created to describe the dose-dependent interactions between miRNA, siRNA, and lenvatinib. The combinatorial effect was evaluated using CompuSyn software, version 1.0 (CompuSyn Inc., Paramus, NJ, USA), and a combination index (CI) was determined, with CI values of <1 being synergistic; = 1 being additive; and > 1 being antagonistic.

### 4.17. Statistical Analysis

All experiments were conducted at least three times on independent days. Data analyses were performed using GraphPad Prism, version 10.1.2 (GraphPad Software, San Diego, CA, USA). For Incucyte assays, results are presented as a mean ± standard error of the mean (SEM), with statistical comparisons made using a paired Student’s *t*-test for comparisons between two groups, and a repeated measures one-way analysis of variance (ANOVA) followed by Dunnett’s post hoc test for multiple group comparisons. For all other experiments, data are represented as a mean ± standard deviation (SD), and analyzed using a two-sided unpaired Student’s t-test or one-way ANOVA followed by Tukey’s post hoc test. A significance threshold was established at *p* < 0.05. Significance levels are indicated as follows: ns = non-significant; * *p* ≤ 0.05; ** *p* ≤ 0.01; *** *p* ≤ 0.001; **** *p* ≤ 0.0001.

## 5. Conclusions

This study revealed that miR-142-3p plays a significant role in modulating resistance to sorafenib and lenvatinib in HCC. MiR -142-3p restores TKI sensitivity partially via targeting YES1 and TWF1, thereby inhibiting YAP1 activation and autophagy, as well as downstream key signaling pathways, including PI3K/AKT, STAT3, and MAPK. Importantly, inhibiting YES1 or TWF1 significantly enhances the sensitivity of cells to lenvatinib and sorafenib in vitro. These findings suggest that targeting these molecules could represent a potentially useful therapeutic strategy for overcoming drug resistance in the treatment of HCC.

## Data Availability

Data can be obtained from the corresponding author upon reasonable request.

## Supplementary Materials

The supporting information can be downloaded at: https://www.mdpi.com/article/10.3390/ijms26094161/s1.
