# Supplementary material for: MicroRNA-142-3p Overcomes Drug Resistance in Hepatocellular Carcinoma by Targeting YES1 and TWF1
Source: Int J Mol Sci. 2025 Apr 27;26(9):4161. doi: 10.3390/ijms26094161 (PMC12071648; doi:10.3390/ijms26094161)
Supplement: Supplementary file 1 [file ijms-26-04161-s001.zip › ijms-3494242-supplementary.pdf]

miR-142-3p Overcomes Drug Resistance in Hepatocellular Carcinoma by Targeting YES1 and TWF1

Supplementary data

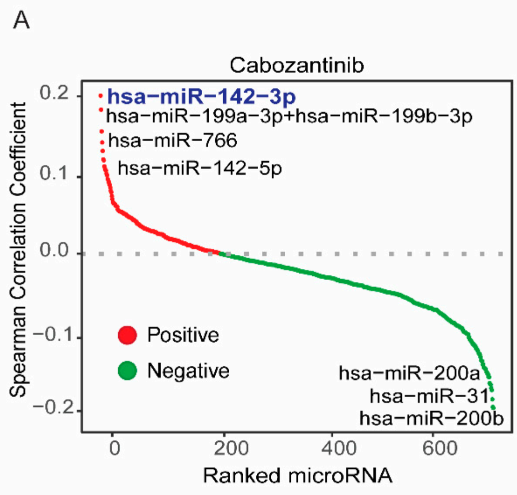

B

Table S1. Basal Expression of miR-142-3p in HCC Panel Cell Lines.

Cell line	Mean Ct_U6	Mean Ct_miR-142-3p
PLC/PRF/5	16.80	32.27
Huh-7	15.23	32.25
SNU449	16.87	32.40
HEP3B	16.46	31.01
SNU475	17.06	29.72
HepG2	16.21	29.62
SNU387	17.02	28.85
**SNU423**	**16.90**	**23.72**

Figure S1. Identification of miR-142-3p as a potential tumor suppressor miRNA in HCC. A) Waterfall plot depicting the correlation between basal miRNA expression and cabozantinib sensitivity in human cancer cell lines (data source: Cancer Cell Encyclopedia and Cancer Therapeutic Response Portal). **B)** Table S1 summarizing the raw Ct values of the basal miR-142-3p expression levels in various HCC cell lines with U6 snRNA as the internal control. Ct = comparative threshold. SNU423 bolded showing the largest expression.

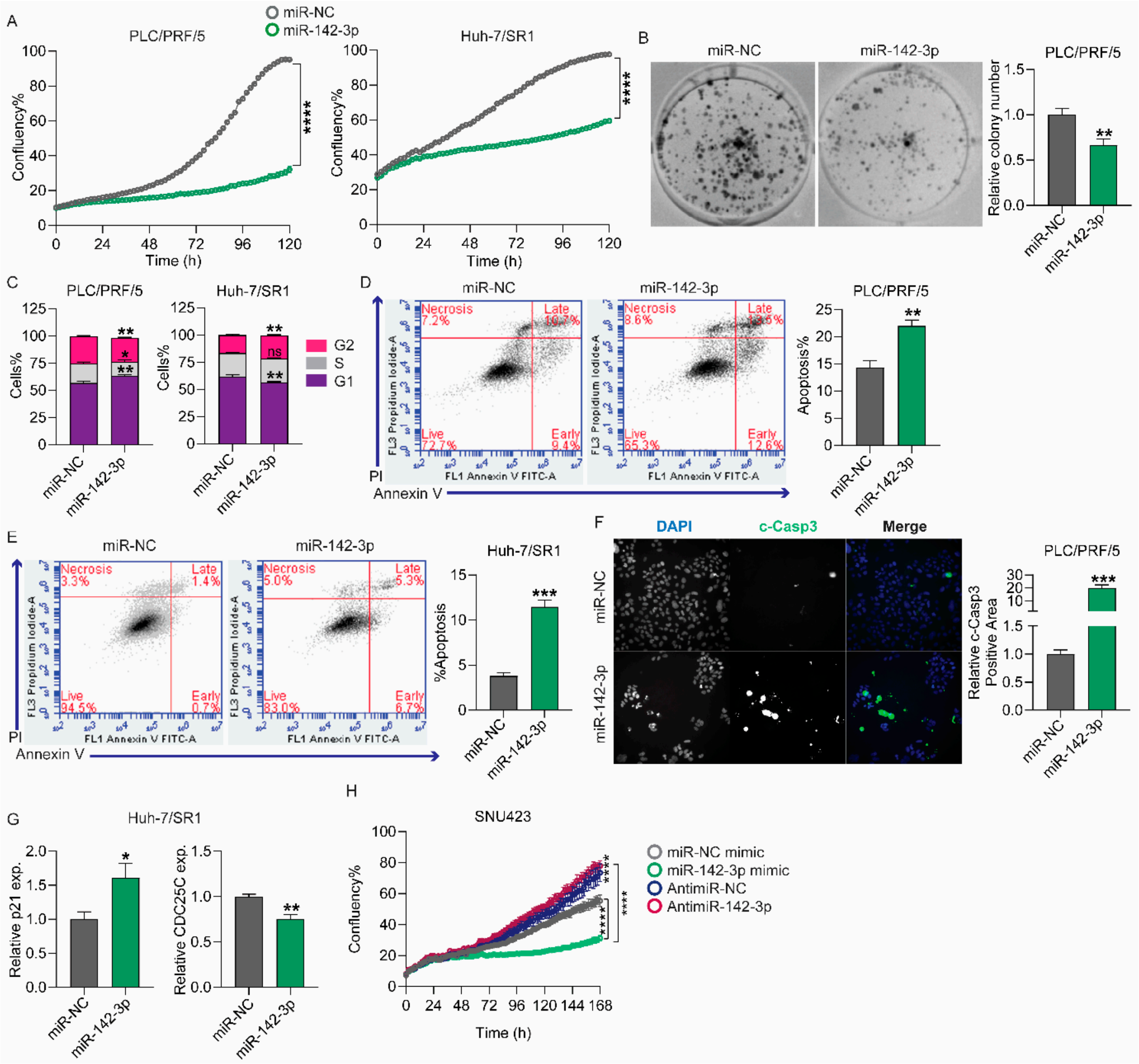

Figure S2. Functional effects of miR-142-3p on proliferation and survival of HCC cells. PLC/PRF/5 and Huh-7/SR1 cells were transfected with 10nM miR-142-3p mimic or miR-NC and assayed as follows: **A)** IncuCyte proliferation assay: time-lapse imaging was conducted at 2 h intervals continuously for up to 120 h. **B)** Effect of miR-142-3p on PLC/PRF/5 colony formation capacity 14 days post transfection. **C)** Flow cytometric analysis of the cell cycle distribution in PLC/PRF/5 and Huh-7/SR1 cells 72 h post-transfection, following propidium iodide staining. **D-E)** The percentage of apoptotic cells was assessed using Annexin V-FITC/propidium iodide staining followed by flow cytometry, with measurements taken 48 h post-transfection for PLC/PRF/5 cells **(D)** and 72 h post-transfection for Huh-7/SR1 cells **(E)**. Bar graph represents the percentage of apoptotic cells. **F)** Apoptosis was investigated by immunofluorescence staining of cleaved-caspase 3 at 48h after transfection in PLC/PRF/5 cells. The magnification is 20X and the image analysis was performed using Thermo Scientific HCS Studio 2.0 Cell Analysis Software on Cellinsight CX7 High Content Analysis System. **G)** RT-qPCR analysis of genes related to cell cycle arrest (p21) and G2/M transition (CDC25C) at 72 h after transfection of Huh-7/SR1 cells. Data were analyzed using the $2^{-\Delta\Delta Ct}$ method with GAPDH as the reference gene and expressed relative to miR-NC. **H)** The IncuCyte proliferation assay of SNU423 cells treated with either a 10 nM miR-142-3p inhibitor or 10 nM miR-142-3p mimic (or their respective negative controls). Error

bars = SEM (for A) and = SD (for B-H); n = 3; Significance levels are indicated as follows: ns = not significant, * $p \leq 0.05$, ** $p \leq 0.01$, *** $p \leq 0.001$, **** $p \leq 0.0001$.

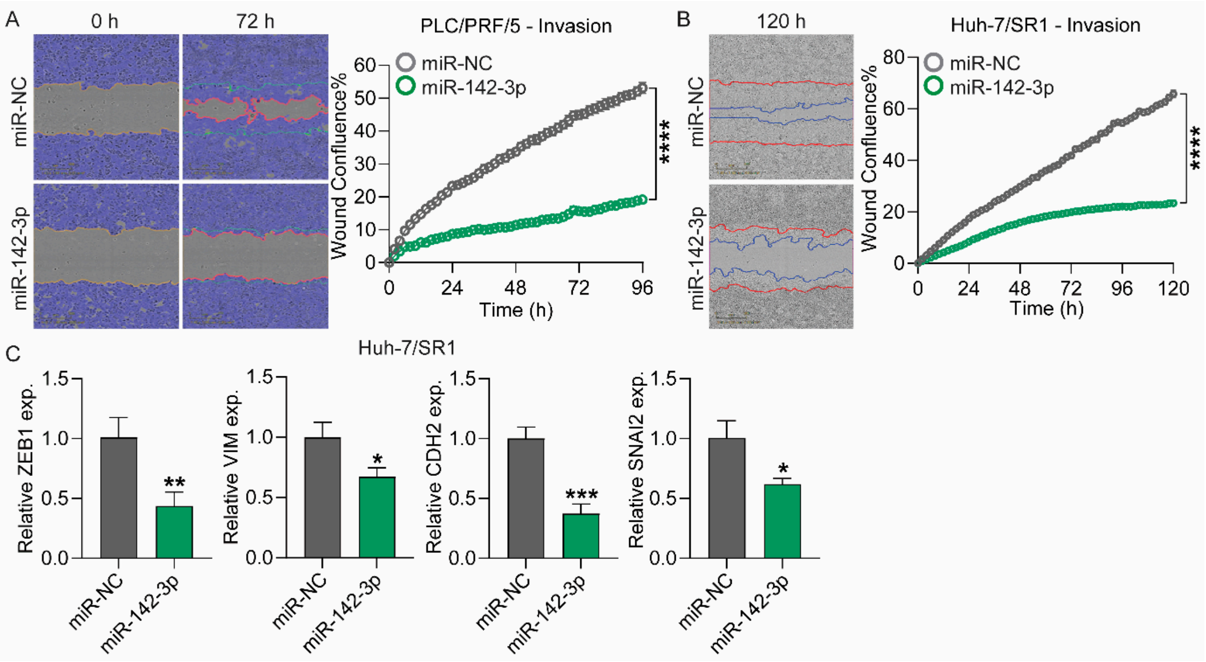

Figure S3. Functional impact of miR-142-3p on HCC cell motility. PLC/PRF/5 and Huh-7/SR1 cells were transfected with 10 nM miR-142-3p mimic or miR-NC and subjected to Matrigel-based wound healing assays to assess invasive capacity. **A)** In PLC/PRF/5 cells, blue lines mark the initial scratch area and red lines indicate the invading cell front. **B)** In Huh-7/SR1 cells, red lines mark the initial scratch area and blue lines indicate the invading front. Live-cell imaging was performed every 2 hours using the IncuCyte ZOOM system until the wound area in control (miR-NC) wells reached full confluence. Wound confluence (%) was quantified over time. Data represent mean ± SEM from six replicate wells. **C)** RT-qPCR analysis of EMT markers in Huh-7/SR1 cells. Data were analyzed using the $2^{-\Delta\Delta Ct}$ method with GAPDH as the reference gene. Mean ± SD values are presented. n = 3 for all experiments. Significance levels are indicated as follows: * $p \leq 0.05$, ** $p \leq 0.01$, *** $p \leq 0.001$, **** $p \leq 0.0001$.

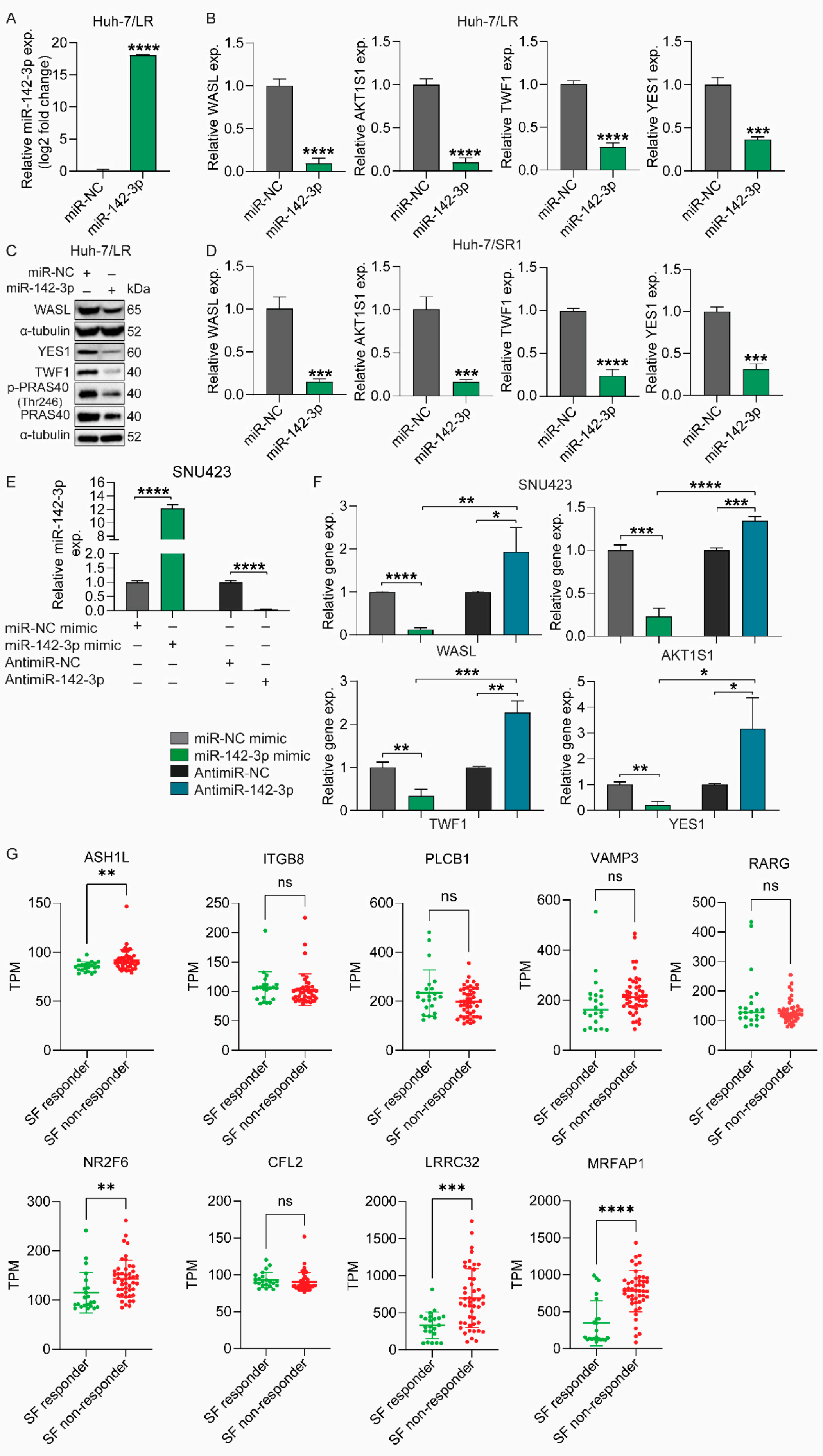

A
Huh-7/LR
Relative miR-142-3p exp. (log2 fold change)
****
miR-NC
miR-142-3p
B
Huh-7/LR
Relative WASL exp.
Relative AKT1S1 exp.
Relative TWF1 exp.
Relative YES1 exp.
C
Huh-7/LR
miR-NC
miR-142-3p
kDa
WASL
α-tubulin
YES1
TWF1
p-PRAS40 (Thr246)
PRAS40
D
Huh-7/SR1
E
SNU423
Relative miR-142-3p exp.
miR-NC mimic
miR-142-3p mimic
AntimiR-NC
AntimiR-142-3p
F
SNU423
Relative gene exp.
WASL
AKT1S1
TWF1
YES1
G
ASH1L
ITGB8
PLCB1
VAMP3
RARG
NR2F6
CFL2
LRRC32
MRFAP1
TPM
ns
SF responder
SF non-responder

Figure S4. Mechanistic pathway analysis and identification of miR-142-3p targets. Huh-7/LR or Huh-7/SR1 cells were transiently transfected with 10 nM miR-142-3p or miR-NC control and assayed as follows: **A)** TaqMan RT-qPCR for miR-142-3p at 24 h post-transfection in Huh-7/LR cells. U6 snRNA served as an internal control. **B)** The mRNA expression levels of miR-142-3p gene targets in Huh-7/LR cells were evaluated using RT-qPCR at 24 h post-transfection. Data were normalized to GAPDH and presented as $2^{-\Delta\Delta Ct}$. **C)** The protein levels of miR-142-3p gene targets in Huh-7/LR cells were assessed 96 h post-transfection. PRAS40 is encoded by the AKT1S1 gene. α-tubulin housekeeping protein. **D)** The impact of miR-142-3p overexpression on the mRNA expression levels of the four identified gene targets was evaluated in Huh-7/SR1 cells at 24 h post-transfection. Data was analyzed using the $2^{-\Delta\Delta Ct}$ method, with GAPDH as the reference gene. SNU423 cells were transiently transfected with 25 nM miRNA mimic or inhibitor for 24 h and analyzed as follows: **E**) miR-142-3p expression levels were measured using a TaqMan miRNA assay. Data was analyzed using the $2^{-\Delta\Delta Ct}$ method with U6 snRNA as the reference gene. **F)** RT-qPCR was performed to assess the mRNA expression of miR-142-3p target genes following its overexpression or depletion. Data were analyzed using the $2^{-\Delta\Delta Ct}$ method with GAPDH as the reference gene. Error bars = standard deviation (SD); n = 3 biological replicates. **G)** Scatter plots display the expression levels of candidate genes potentially associated with drug resistance, measured in transcripts per million (TPM), in sorafenib responders and non-responders from the BIOSTORM HCC cohort. Each dot represents an individual patient sample. Horizontal bars indicate the mean ± SD. P-values reflect the statistical significance of differential expression between the two groups, determined using an unpaired two-tailed t-test. Significance levels are indicated as follows: ns: not significant; * $p \leq 0.05$, ** $p \leq 0.01$, *** $p \leq 0.001$, and **** $p \leq 0.0001$.

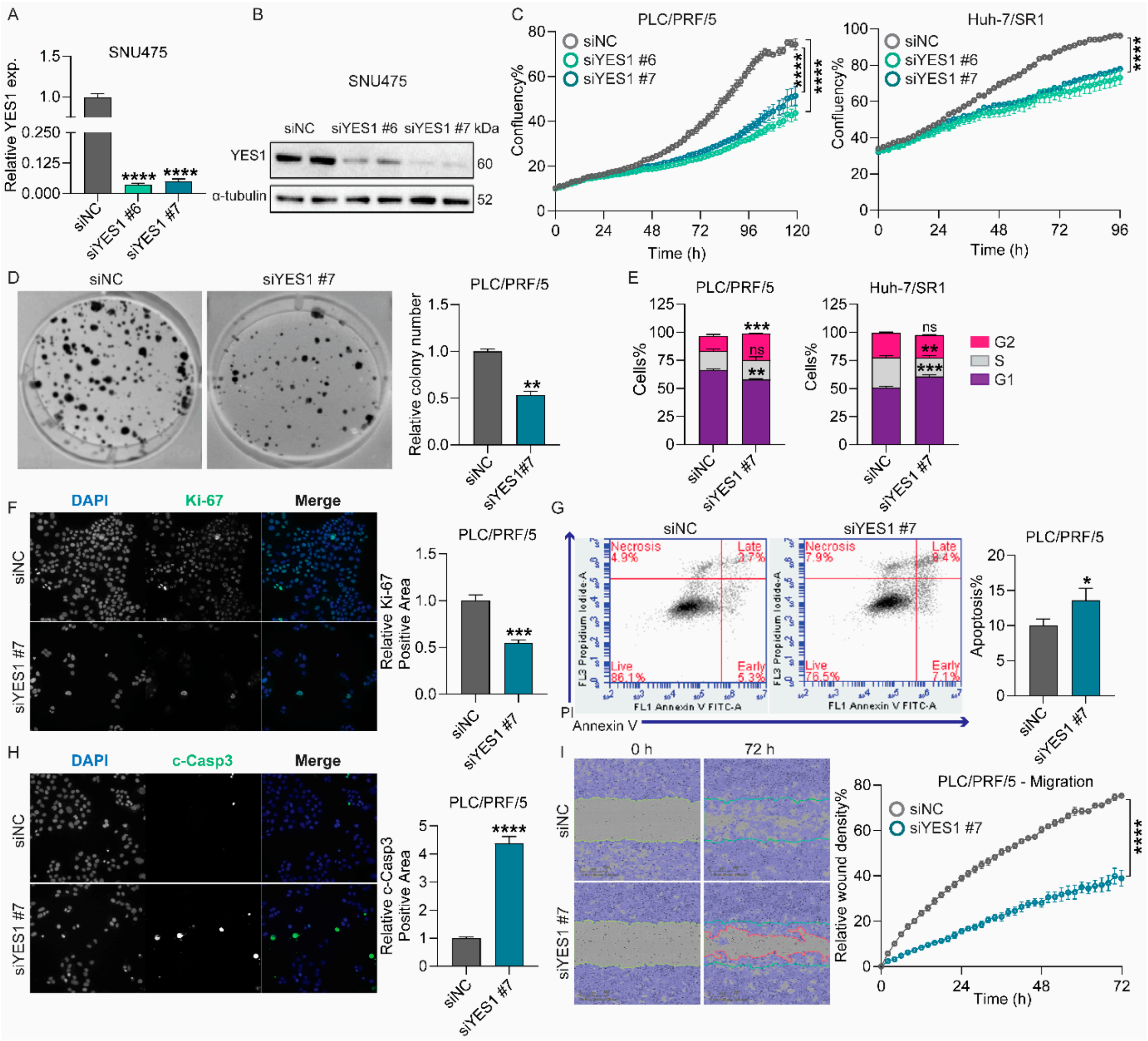

Figure S5. The effect of YES1 knockdown on HCC cell survival and motility. SNU475, PLC/PRF/5 or Huh-7/SR1 cells were transiently transfected with 10 nM siYES1 #6, siYES1 #7 or siNC control and assayed as follows: **A)** RT-qPCR analysis was performed to validate YES1 knockdown in SNU475 cells, 24 h post-transfection. Data were analyzed using the $2^{-\Delta\Delta Ct}$ method, with GAPDH serving as the reference gene. **B)** Western blot analysis to evaluate the knockdown efficiency of YES1 at protein level in SNU475 cells 96 h post-transfection. α-tubulin was used as an internal control (housekeeping protein). **C)** The IncuCyte proliferation assay displays the effect of YES1 knockdown on PLC/PRF/5 and Huh-7/SR1 cells compared with the siNC control. Time-lapse imaging was conducted at 2 h intervals continuously for up to 120 h. **D)** Effect of YES1 knockdown on the colony formation capacity of PLC/PRF/5 cells 14 days post-transfection. **E)** Flow cytometric analysis of the cell cycle distribution in PLC/PRF/5 and Huh-7/SR1 cells 72 h post-transfection, following propidium iodide staining. **F)** Immunofluorescence analysis of Ki-67 expression at 72h post-transfection in PLC/PRF/5 cells. The magnification is 20X and the image analysis was performed using Thermo Scientific HCS Studio 2.0 Cell Analysis Software on Cellinsight CX7 High Content Analysis System. **G)** YES1 downregulation-associated apoptosis was investigated by Annexin V-FITC/propidium iodide staining and flow cytometry 48 h after transfection. **H)** Immunofluorescence analysis of cleaved caspase 3 (c-Casp3) 48h post-transfection of PLC/PRF/5 cells to validate apoptosis induction. Imaging is as per above (Fig. S5F). **I)** Wound healing assay to study the migration ability of PLC/PRF/5 cells following siYES1 transfection.

The relative wound density (%) within the scratch area was calculated based on phase-contrast images using the IncuCyte ZOOM software. Blue lines represent initial scratches. Error bars = SD; n = 3; Significance levels are indicated as follows: ns = not significant, * $p \leq 0.05$, ** $p \leq 0.01$, *** $p \leq 0.001$, **** $p \leq 0.0001$.

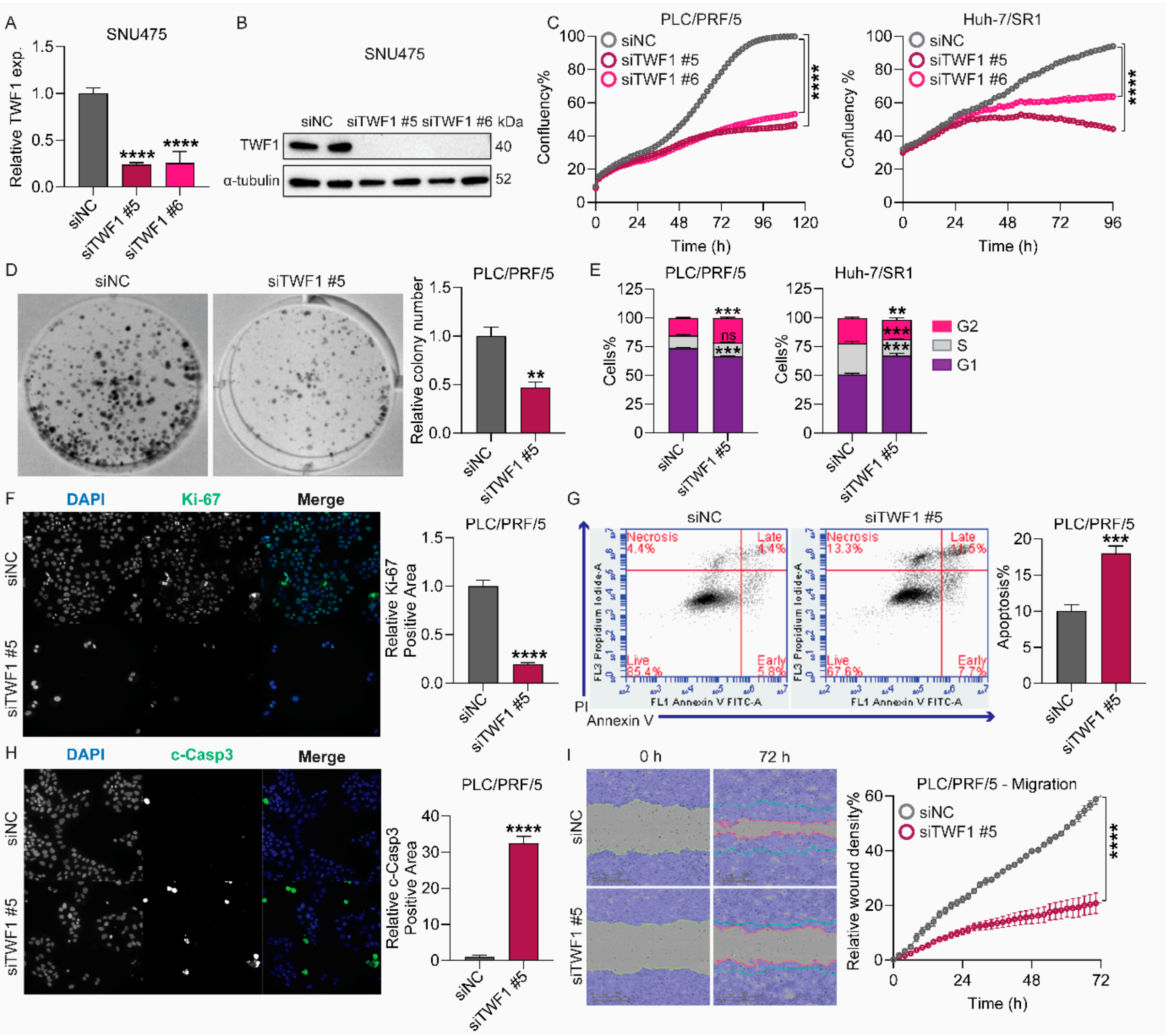

Figure S6. Effects of TWF1 knockdown on HCC cell survival and motility. SNU475, PLC/PRF/5 or Huh-7/SR1 cells were transfected with 10 nM siTWF1 #5 or #6, or a siNC control and assayed as follows: **A)** RT-qPCR analysis was performed to validate TWF1 knockdown in SNU475 cells 24 h after transfection. Data were analyzed using the $2^{-\Delta\Delta Ct}$ method, with GAPDH as the reference gene. **B)** Western blot analysis to evaluate TWF1 protein knockdown efficiency in SNU475 cells 96 h post-transfection. α-tubulin was used as an internal control (housekeeping protein). **C)** IncuCyte proliferation assay using time-lapse imaging at 2 h intervals continuously for up to 120 h. **D)** Effect of TWF1 knockdown on the colony formation capacity of PLC/PRF/5 cells 14 days post-transfection. **E)** Flow cytometric analysis of the cell cycle distribution in PLC/PRF/5 and Huh-7/SR1 cells transfected for

72 h, following propidium iodide staining. **F)** Immunofluorescence analysis of Ki-67 expression 72 h post-transfection of PLC/PRF/5 cells. The magnification is 20X and the image analysis was performed using Thermo Scientific HCS Studio 2.0 Cell Analysis Software on Cellinsight CX7 High Content Analysis System. **G)** TWF1 downregulation-associated apoptosis was investigated by Annexin V-FITC/propidium iodide staining and flow cytometry in PLC/PRF/5 cells after 48h transfection. **H)** Immunofluorescence analysis of cleaved caspase 3 (c-Casp3) 48 h post-transfection of PLC/PRF/5 cells to validate apoptosis induction. Imaging analysis is as per Fig. S6F above. **I)** The effect of TWF1 knockdown on the migration ability of PLC/PRF/5 cells was evaluated using a wound healing assay. The relative wound density (%) within the scratch area was calculated based on phase-contrast images using IncuCyte ZOOM software. Blue lines represent initial scratches. Error bars = SD (A-B, D-H) or SEM (C and I), n = 3; Significance levels are indicated as follows: ns = non-significant, ** $p \leq 0.01$, *** $p \leq 0.001$, **** $p \leq 0.0001$.

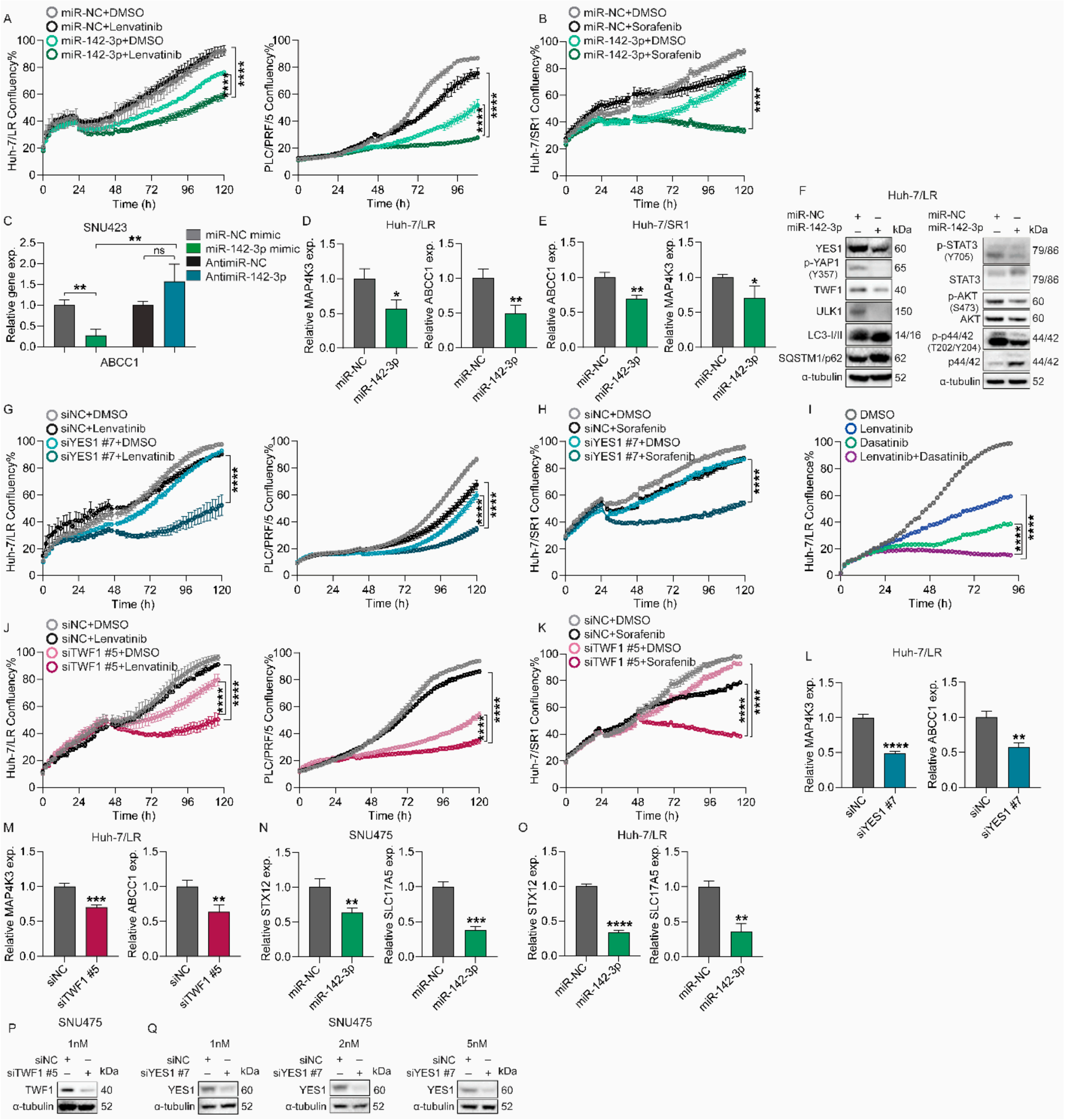

Figure S7. Role of miR-142-3p and its gene targets in modulating TKI sensitivity in HCC cells. A-B) IncuCyte proliferation assay of combinational treatment of Huh-7/LR, PLC/PRF/5, or Huh-7/SR1 cells with 5 nM transfection of miR-142-3p (or control miRNA) (for 48 h), followed by 10 µM lenvatinib (Huh-7/LR and PLC/PRF/5) or 10 µM sorafenib (Huh-7/SR1). Time-lapse imaging was conducted at 2 h intervals continuously for up to 120 h. **C-E)** RT-qPCR analysis 24 h post-transfection in: **C)** SNU423 cells transiently transfected with 25 nM miR-142-3p or antimiR-142-3p (and the relevant negative controls), or **D-E)** Huh-7/LR and Huh-7/SR1 cells transiently transfected with 10 nM miR-142-3p or miR-NC. Data were analyzed using the $2^{-\Delta\Delta Ct}$ method, with GAPDH as the reference gene. **F)** Drug resistance-associated pathway western blot analysis in Huh-7/LR cells following overexpression of miR-142-3p (10 nM) for 96 h. α-tubulin was used as an internal control (housekeeping protein). **G-H)** Evaluation of the synergistic potential of combining YES1 knockdown with lenvatinib or sorafenib on HCC cell proliferation using the following concentrations: Huh-7/LR cells: siYES1 #7 (2.5 nM) and lenvatinib (5 µM); PLC/PRF/5 cells: siYES1 #7 (5 nM) and lenvatinib (10 µM) **(G)**; Huh-7/SR1 cells:

siYES1 #7 (2.5 nM) and sorafenib (5 μM) **(H)**. **I)** Assessment of the synergistic effects of dasatinib (IC25 dose: 16 μM) and lenvatinib (IC50 dose: 24.73 μM) on Huh-7/LR cell proliferation using the IncuCyte assay. **J-K)** Evaluation of the synergistic potential of combining TWF1 knockdown with lenvatinib or sorafenib using the following concentrations: Huh-7/LR cells: siTWF1 #5 (1 nM) and lenvatinib (10 μM); PLC/PRF/5 cells: siTWF1 #5 (5 nM) and lenvatinib (10 μM) **(J)**; Huh-7/SR1 cells: siTWF1 #5 (1 nM) and sorafenib (10 μM) **(K)**. **L-M)** RT-qPCR analysis of MAP4K3 and ABCC1 mRNA expression in Huh-7/LR cells transfected with 10nM siYES1 #7 **(L)** or siTWF1 #5 **(M)**. Data were analyzed using the $2^{-\Delta\Delta Ct}$ method with GAPDH as the reference gene. **N-O)** RT-qPCR analysis was performed to assess STX12 and SLC17A5 mRNA expression levels in SNU475 **(N)** and Huh-7/LR cells **(O)** following miR-142-3p overexpression using 10 nM miRNA mimic for 24 h compared to miR-NC. Data were analyzed using the $2^{-\Delta\Delta Ct}$ method, with HPRT as the reference gene. **P-Q)** Validation of gene knockdown efficiency at the protein level in SNU475 cells transfected for 96 h with 1 nM siTWF1 #5 **(P)**, 1, 2, or 5 nM of siYES1 #7 **(Q)**, compared to siNC as a control. These low concentrations of siRNAs were specifically selected for combination therapy with Lenvatinib or sorafenib. Error bars = SEM (A-B, G-K) or SD (C-E, M-O); n = 3; Significance levels are indicated as follows: * $p \leq 0.05$, ** $p \leq 0.01$, *** $p \leq 0.001$, **** $p \leq 0.0001$.

Table S2. List of reagents used in this study.

Reagent	Manufacturer	Catalog #
**Cell culture**		
RPMI 1640	Gibco	11875093
MEM	Gibco	11095080
low-glucose DMEM	Gibco	11885084
FBS	Sigma	F9423
Sodium pyruvate	Gibco	11360070
Non-essential amino acids	Gibco	11140050
GlutaMax	Gibco	35050061
HEPES	Gibco	15630130
**Primers**		
WASL	QuantiTect-Qiagen	GeneGlobe ID - QT00024416<br>Catalog No. - 249900
TWF1	QuantiTect-Qiagen	GeneGlobe ID - QT00036883<br>Catalog No. - 249900
YES1	QuantiTect-Qiagen	GeneGlobe ID - QT00037940<br>Catalog No. - 249900
MAP4K3	QuantiTect-Qiagen	GeneGlobe ID - QT00081557<br>Catalog No. - 249900
ABCC1	QuantiTect-Qiagen	GeneGlobe ID - QT00061159<br>Catalog No. - 249900
CDC25C	QuantiTect-Qiagen	GeneGlobe ID - QT00000350<br>Catalog No. - 249900
CDH2	QuantiTect-Qiagen	GeneGlobe ID - QT00063196<br>Catalog No. - 249900
VIM	QuantiTect-Qiagen	GeneGlobe ID - QT00095795<br>Catalog No. - 249900

<table>
<tr><td>ZEB1</td><td>QuantiTect-Qiagen</td><td>GeneGlobe ID - QT00020972
Catalog No. - 249900</td></tr>
<tr><td>ZEB2</td><td>QuantiTect-Qiagen</td><td>GeneGlobe ID - QT00008554
Catalog No. - 249900</td></tr>
<tr><td>HPRT</td><td>QuantiTect-Qiagen</td><td>GeneGlobe ID - QT00059066
Catalog No. - 249900</td></tr>
<tr><td>GAPDH</td><td>QuantiTect-Qiagen</td><td>GeneGlobe ID - QT00079247
Catalog No. - 249900</td></tr>
<tr><td>AKT1S1</td><td>Sigma</td><td>F: GCATCTGTCCCGTTTCCCGAT
R: AGAACTCAGCGAGCCAATCCC</td></tr>
<tr><td>p21</td><td>Sigma</td><td>F: AATAATGCCGCCGCCTCTTC
R: TTAATTGTTCCATCGCTCACG</td></tr>
<tr><td>SLUG</td><td>Sigma</td><td>F: ATACAGCCCCATCACTGTGT
R: GACTCACTCGCCCCAAAGATGA</td></tr>
<tr><td colspan="3">Antibodies</td></tr>
<tr><td>WASL</td><td>Cell Signaling</td><td>4848</td></tr>
<tr><td>p-PRAS40 (T246)</td><td>Cell Signaling</td><td>2997</td></tr>
<tr><td>PRAS40</td><td>Cell Signaling</td><td>2610</td></tr>
<tr><td>TWF1</td><td>Cell Signaling</td><td>8461</td></tr>
<tr><td>YES1</td><td>Cell Signaling</td><td>65890</td></tr>
<tr><td>VIM</td><td>Cell Signaling</td><td>3390</td></tr>
<tr><td>Anti-PARP (214/215) cleavage site Antibody</td><td>Merck Millipore</td><td>AB3565</td></tr>
<tr><td>GPX4</td><td>Cell Signaling</td><td>59735</td></tr>
<tr><td>P21</td><td>Cell Signaling</td><td>2947</td></tr>
<tr><td>p-p44/42 MAPKL (T202/Y204)</td><td>Cell Signaling</td><td>9101</td></tr>
<tr><td>P44/42 Erk1/2 MAPK</td><td>Cell Signaling</td><td>9102</td></tr>
<tr><td>α-tubulin</td><td>Abcam</td><td>ab7291</td></tr>
<tr><td>ULK1</td><td>Cell Signaling</td><td>8054</td></tr>
<tr><td>p-STAT3 (Y705)</td><td>Cell Signaling</td><td>9131</td></tr>
<tr><td>STAT3</td><td>Cell Signaling</td><td>9132</td></tr>
<tr><td>p-YAP (Y357)</td><td>Abcam</td><td>ab62751</td></tr>
<tr><td>p-AKT (S473)</td><td>Abcam</td><td>ab8932</td></tr>
<tr><td>AKT</td><td>Cell Signaling</td><td>9272</td></tr>
<tr><td>SQSTM1/p62</td><td>Cell Signaling</td><td>5114</td></tr>
<tr><td>Ki67</td><td>Abcam</td><td>ab15580</td></tr>
<tr><td>Cleaved-caspase 3</td><td>Cell Signaling</td><td>9661</td></tr>
<tr><td>ECL™Anti-rabbit IgG, horseradish peroxidase (HRP)-conjugated</td><td>Cytiva</td><td>NA934V</td></tr>
<tr><td>ECL™Anti-mouse IgG, horseradish peroxidase (HRP)-conjugated</td><td>Cytiva</td><td>NA931V</td></tr>
<tr><td>Goat anti-Rabbit IgG (H+L) Cross-Adsorbed Secondary Antibody, Alexa Fluor™ 488</td><td>Thermo Fisher Scientific</td><td>A-11008</td></tr>
<tr><td colspan="3">siRNAs</td></tr>
<tr><td>Hs_PTK9_6</td><td>Qiagen</td><td>GeneGlobe Id: SI02621948|S1
Catalog Number: 1027417</td></tr>
<tr><td>Hs_PTK9_5</td><td>Qiagen</td><td>GeneGlobe Id: SI02223025|S1</td></tr>
</table>

		Catalog Number: 1027417
Hs_PTK9_9	Qiagen	GeneGlobe Id: SI03030111|S1<br>Catalog Number: 1027417
Hs_YES1_5	Qiagen	GeneGlobe Id: SI00302218|S1<br>Catalog Number: 1027417
Hs_YES1_6	Qiagen	GeneGlobe Id: SI02223935|S1<br>Catalog Number: 1027417
Hs_YES1_7	Qiagen	GeneGlobe Id: SI02223942|S1<br>Catalog Number: 1027417
Negative Control siRNA	Qiagen	1022076
**miRNAs**		
hsa-miR-142-3p Ambion® Pre-miR™ miRNA Precursor	Thermo Fisher Scientific	Assay ID: PM10398
miRIDIAN microRNA Mimic Negative Control #2	Dharmacon	CN-002000-01-05
hsa-miR-142-3p Ambion® Anti-miR™ miRNA Inhibitor	Thermo Fisher Scientific	Assay ID: AM10398
Anti-miR™ miRNA Inhibitor Negative Control #1	Thermo Fisher Scientific	AM17010
**Drugs**		
Sorafenib	Selleckchem	S7397
Lenvatinib	Selleckchem	S5240
Dasatinib	Selleckchem	S1021
Mitomycin C	Merck	M4287
DMSO	Merck	D8418
**Other reagents**		
Lipofectamine™ 2000 Transfection Reagent	Thermo Fisher Scientific	11668019
Rhodamine Phalloidin	Biotium	00027
LysoTracker™ Deep Red	Thermo Fisher Scientific	L12492
DQ™ Red BSA	Thermo Fisher Scientific	D12051
Hoechst 33342	Sigma	14533
4′,6-diamidino-2-phenylindole (DAPI)	Sigma	D8417
RNeasy Mini Kit	Qiagen	74104
RNA 6000 Nano Kit	Agilent	5067-1511
Qubit RNA IQ Assay Kit	Thermo Fisher Scientific	Q33222
TRIzol™ Reagent	Thermo Fisher Scientific	15596026
QIAprep Spin Miniprep Kit	Qiagen	27104
Matrigel	Corning	356234
Paraformaldehyde (PFA)	Sigma	47608
Triton™ X-100	Merck	9036-19-5
Propidium iodide	Sigma	81845
PureLink™ RNase A (20 mg/mL)	Thermo Fisher Scientific	12091021
FITC Annexin V Apoptosis Detection Kit I	BD Pharmingen	556547
pEZX-MT06-TWF1-WT	GeneCopoeia	HmiT067212-MT06
pEZX-MT06-TWF1-MUT	GeneCopoeia	CSHmi149033-

		MT06-01-10
pEZX-MT06-YES1-WT	GeneCopoeia	HmiT149033-MT06
pEZX-MT06-YES1-MUT	GeneCopoeia	CSHmiT067212-MT06-01-10
CellTiter 96® AQueous One Solution Cell Proliferation Assay (MTS)	Promega	G3582
Luciferasae Assay kit	Promega	E1500
QuantiTect Reverse Transcription Kit	Qiagen	205311
hsa-miR-142-3p TaqMan™ MicroRNA Assay	Thermo Fisher Scientific	Assay ID: 000464
U6 snRNA TaqMan™ microRNA Control Assay	Thermo Fisher Scientific	Assay ID: 001973
TaqMan™ MicroRNA Reverse Transcription Kit	Applied Biosystems™	4366596
TaqMan™ Universal Master Mix II, no UNG	Applied Biosystems™	4440043
SensiMix™ SYBR® Hi-ROX Kit	Meridian Biosience Bioline	QT605-05
Cell Lysis Buffer (10X)	Cell signaling	9803
PhosSTOP™	Roche	04906837001
cOmplete™, Mini, EDTA-free Protease Inhibitor Cocktail	Roche	11836170001
Phenylmethylsulfonyl fluoride (PMSF)	Sigma	10837091001
Bio-Rad Protein Assay Dye Reagent Concentrate	Bio-Rad	5000006
PVDF Western Blotting Membranes	Merck	3010040001
BSA	Cell signaling	9998S
Bis-Tris Mini Protein Gels, 4–12%, 1.0–1.5 mm	Thermo Fisher Scientific	NP0321BOX
NuPAGE™ Transfer Buffer (20X)	Thermo Fisher Scientific	NP0006
NuPAGE™ Sample Reducing Agent (10X)	Thermo Fisher Scientific	NP0004
NuPAGE™ LDS Sample Buffer (4X)	Thermo Fisher Scientific	NP0007
NuPAGE™ Antioxidant	Thermo Fisher Scientific	NP0005
Immobilon Crescendo Western HRP substrate	Merck Millipore	WBLUR0100
Precision Plus Protein™ Kaleidoscope™ Prestained Protein Standards	Bio-Rad	1610375

Table S3. List of 80 significantly downregulated genes by miR-142-3p treatment in SNU475 cells.

Gene name	Gene type	P value	FDR step up	Fold change	Log2 Fold change
UTP14C	protein coding	3.24E-03	1.52E-02	-6.91	6.91
**WASL**	protein coding	0.00E+00	0.00E+00	-5.82	5.82
AC092647.5	protein coding	1.32E-04	8.73E-04	-4.3	4.3
AC141586.3	lncRNA	5.45E-06	4.70E-05	-3.7	3.7
ZNF559-ZNF177	protein coding	7.12E-05	5.00E-04	-3.47	3.47
AC010422.6	protein coding	8.66E-04	4.77E-03	-3.42	3.42
LRRC32	protein coding	1.22E-143	3.61E-141	-3.36	3.36
MANBAL	protein coding	6.47E-212	3.27E-209	-3.34	3.34
LINC02649	lncRNA	8.36E-03	3.46E-02	-3.1	3.1
CFL2	protein coding	9.78E-253	7.00E-250	-3.09	3.09
NEFH	protein coding	1.55E-15	3.45E-14	-3.08	3.08
TSEN34	protein coding	3.01E-84	4.30E-82	-3.07	3.07
NR2F6	protein coding	7.49E-65	7.75E-63	-2.97	2.97
**AKT1S1**	protein coding	2.77E-172	1.11E-169	-2.94	2.94
RARG	protein coding	1.54E-100	2.72E-98	-2.93	2.93
BOD1	protein coding	7.00E-121	1.67E-118	-2.87	2.87
VAMP3	protein coding	0.00E+00	0.00E+00	-2.87	2.87
PNPLA7	protein coding	1.15E-02	4.53E-02	-2.84	2.84
TNFSF14	protein coding	1.15E-02	4.52E-02	-2.84	2.84
SLC49A4	protein coding	1.78E-23	5.85E-22	-2.8	2.8
SLC17A5	protein coding	7.99E-222	4.57E-219	-2.79	2.79
PCDHGA3	protein coding	1.67E-03	8.54E-03	-2.71	2.71
REPS2	protein coding	3.91E-03	1.80E-02	-2.67	2.67
FAM114A1	protein coding	7.410985e-323	8.480637e-320	-2.66	2.66
RPL5P29	processed pseudogene	6.63E-03	2.83E-02	-2.63	2.63
ADAM1B	unitary pseudogene	3.27E-03	1.53E-02	-2.62	2.62
NKILA	lncRNA	3.27E-03	1.54E-02	-2.62	2.62
TWF1P1	processed pseudogene	5.21E-55	4.36E-53	-2.55	2.55
ELFN2	lncRNA ǀ protein coding	4.06E-05	2.99E-04	-2.52	2.52
TNRC18P3	unprocessed pseudogene	4.88E-04	2.84E-03	-2.51	2.51
IL6ST	protein coding	0.00E+00	0.00E+00	-2.5	2.5
**TWF1**	protein coding	7.12E-279	6.11E-276	-2.5	2.5
PPP1R37	protein coding	1.89E-103	3.49E-101	-2.46	2.46
INPP5A	protein coding	1.23E-25	4.47E-24	-2.45	2.45
AL049629.2	protein coding	4.62E-10	6.76E-09	-2.4	2.4
**YES1**	protein coding	7.30E-214	3.80E-211	-2.39	2.39
GJD3	protein coding	2.60E-06	2.38E-05	-2.36	2.36
RTL8A	protein coding	2.62E-111	5.48E-109	-2.36	2.36
SSPN	protein coding	1.72E-28	6.98E-27	-2.35	2.35
C20orf194	protein coding	3.53E-165	1.35E-162	-2.33	2.33
PLCB1	protein coding	7.18E-03	3.03E-02	-2.31	2.31
FAM222B	protein coding	2.58E-111	5.46E-109	-2.3	2.3
ITGB8	protein coding	1.49E-08	1.86E-07	-2.3	2.3
PAFAH1B2	protein coding	1.35E-163	5.03E-161	-2.28	2.28

SC5D	protein coding	1.01E-274	8.25E-272	-2.26	2.26
CLDN12	protein coding	1.94E-65	2.05E-63	-2.25	2.25
COPS7A	protein coding	3.64E-81	4.92E-79	-2.23	2.23
RHOBTB3	protein coding	0.00E+00	0.00E+00	-2.23	2.23
ZBTB41	protein coding	0.00E+00	0.00E+00	-2.22	2.22
ZNF467	protein coding	4.07E-05	3.00E-04	-2.22	2.22
CYP7B1	protein coding	9.55E-03	3.88E-02	-2.19	2.19
AC008758.1	protein coding	9.56E-03	3.88E-02	-2.17	2.17
DCUN1D4	protein coding	2.95E-123	7.22E-121	-2.15	2.15
EPN1	protein coding	2.81E-216	1.55E-213	-2.15	2.15
ASH1L	protein coding	6.31E-304	6.77E-301	-2.14	2.14
STXBP4	protein coding	3.90E-20	1.12E-18	-2.14	2.14
DBP	protein coding	1.36E-15	3.03E-14	-2.12	2.12
TMTC3	protein coding	7.53E-92	1.17E-89	-2.12	2.12
PRRG2	protein coding	5.80E-05	4.15E-04	-2.11	2.11
CLIC4P3	processed pseudogene	7.30E-04	4.08E-03	-2.1	2.1
FNTB	protein coding	3.89E-17	9.57E-16	-2.1	2.1
DDIT4	protein coding	1.78E-92	2.81E-90	-2.09	2.09
ITPR3	protein coding	0.00E+00	0.00E+00	-2.09	2.09
ZFYVE28	protein coding	8.16E-03	3.39E-02	-2.09	2.09
SKP2	protein coding	2.07E-67	2.28E-65	-2.08	2.08
HOXD1	protein coding	9.85E-03	3.98E-02	-2.07	2.07
SLC2A12	protein coding	2.16E-30	9.37E-29	-2.07	2.07
STX12	protein coding	1.85E-121	4.47E-119	-2.07	2.07
WIZ	protein coding	1.10E-65	1.18E-63	-2.07	2.07
AC103591.4	lncRNA	7.43E-03	3.12E-02	-2.06	2.06
MRFAP1	protein coding	0.00E+00	0.00E+00	-2.05	2.05
AL354740.1	lncRNA	3.26E-10	4.83E-09	-2.04	2.04
FLNC	protein coding	2.37E-194	1.10E-191	-2.04	2.04
FRK	protein coding	3.43E-38	1.93E-36	-2.03	2.03
GOLGA4	protein coding	3.61E-77	4.56E-75	-2.03	2.03
ARL2	protein coding	1.39E-67	1.53E-65	-2.02	2.02
GDPD1	protein coding	4.15E-05	3.05E-04	-2.02	2.02
SIK1B	protein coding	2.11E-22	6.63E-21	-2.02	2.02
AC073073.2	lncRNA	2.66E-04	1.65E-03	-2.01	2.01
RYR1	protein coding	1.08E-02	4.31E-02	-2.01	2.01

Table S4. Summary of chemoresistance associations for the 27 overlapping gene.

Gene	Association with Chemoresistance?	Cancer Type & Resistant Drug	Evidence Type	References
**WASL (N-WASP)**	**Not clearly reported.** Overexpression is linked to metastasis and hypoxia, but no direct evidence of chemoresistance.	(N/A) – Pancreatic cancer data suggest WASL (N-WASP) is oncogenic and its loss induces senescence. Hypoxia elevates N-WASP, contributing to a resistant tumor environment .	Preclinical (mouse models, cell studies)	[1, 2]
**LRRC32 (GARP)**	**Yes – promotes therapy resistance.** Overexpressed GARP activates TGF-β, aiding tumor immune evasion and resistance.	Bone sarcoma – **Chemotherapy & radiotherapy** resistance; **Immune Checkpoint Inhibitors (ICI)** resistance.	*In vitro* and *in vivo* (silencing studies); Clinical correlation.	[3]
**MANBAL**	**No known association.**	–	– (No data)	–
**CFL2 (Cofilin-2)**	**Limited evidence;** not directly linked to chemoresistance. Some association with treatment resistance.	Nasopharyngeal carcinoma – **radioresistance**; prostate cancer – tumor progression (non-drug specific)	Clinical correlation; suggested functional relevance via SOX2-OT/miR-369-3p/CFL2 axis	[4, 5]
**TSEN34**	**No known association.**	–	– (No data)	–
**NR2F6**	**Yes – confers resistance.** Overexpression of NR2F6 (an orphan nuclear receptor) promotes chemoresistance and immune evasion.	Ovarian cancer – **Cisplatin**; Melanoma – **Immunotherapy** (anti–PD1).	*In vitro* and tumor xenograft studies; Patient samples.	[6, 7]
**AKT1S1 (PRAS40)**	**Yes – promotes drug resistance.** Overexpressed PRAS40 (AKT1S1) enhances survival signaling.	HNSCC - EGFR inhibitors	*In vitro* (knockdown/overexpression in cell lines).	[8]
**RARG**	**Yes – linked to multi-drug resistance.** An oncogenic RARγ can drive therapy resistance via Wnt signaling.	Ovarian cancer –Acute myeloid leukemia and Acute promyelocytic leukemia	*In vitro* drug sensitivity assays; Patient tumor analyses.	[9, 10]
**BOD1**	**Partial evidence – enhances resistance**	Lung cancer (NSCLC) – **Radiation** (and	*In vitro* radiation sensitivity assays.	[11]

	**to stress.** Overexpression of BOD1 (a mitotic protein) confers resistance to radiation; indirectly linked to chemo via autophagy.	possibly chemo via autophagy).		
**VAMP3**	**Yes – promotes chemoresistance via autophagy and drug efflux.** Overexpressed VAMP3 (vesicle SNARE) enhances autophagy and exosome-mediated drug sequestration.	Breast cancer – **Doxorubicin, Paclitaxel**; Triple-negative breast cancer (TNBC) – **Paclitaxel**.	*In vitro* resistant cell line studies; Functional assays.	[12, 13]
**FAM114A1**	**No known association.**	–	– (No data)	–
**TWF1 (Twinfilin-1)**	**Yes – enhances chemoresistance.** High TWF1 promotes survival under chemotherapy and possibly limits immune response.	Breast cancer – **Paclitaxel**; Lung cancer – **Various** (chemo and immune context).	*In vitro* (drug-response assays); Bioinformatic and clinical correlations.	[14-16]
**PPP1R37**	**No known association.**	–	– (No data)	–
**INPP5A**	**No clear association (likely tumor-suppressive).** INPP5A is often downregulated in cancer rather than overexpressed.	Melanoma, SCC – loss correlates with progression.	Clinical observations.	[17]
**YES1**	**Yes – well-established.** Overexpression of YES1 (Src-family kinase) is a known driver of resistance to multiple targeted therapies and some chemotherapies.	Lung cancer – **EGFR TKI (erlotinib)**; Breast cancer – **HER2 therapies (e.g. T-DM1)**; General – various TKIs and chemo.	Clinical resistance cases (amplifications in patients); *In vitro* overexpression studies.	[18-20]
**C20orf194**	**No known association.** (Uncharacterized gene)	–	– (No data)	–
**PLCB1**	**Yes – promotes therapy resistance.** Overactive PLCβ1 triggers pro-survival signaling (PI3K–AKT),	Triple-negative breast cancer – **Radio- & immune-resistance**; Glioma – **Chemoresistance** (hypoxia-induced).	*In vitro* (radiation-selected cell lines, knockdown); Patient tumors.	[21-23]

	leading to drug and radio-resistance.			
**ITGB8**	**Yes – enhances resistance.** Integrin β8 overexpression leads to drug resistance and an immunosuppressive microenvironment via TGF-β activation and YBX1 signaling.	Liver cancer cell line – **Gefitinib (EGFR TKI)**; Bladder cancer – **Mitomycin C, Hydroxycamptothecin**; Pancreatic cancer – **Radio/chemoresistanc**.	*In vitro* resistant cell model; Tumor xenografts; Clinical samples.	[24, 25]
**PAFAH1B2**	**No direct evidence.** (Oncogenic in some cancers, but role in drug resistance not reported.)	Pancreatic cancer (HIF1α-induced EMT and invasion); Ovarian cancer (tumorigenicity and migration reduced upon knockdown)	*In vivo* (correlative).	[26, 27]
**RHOBTB3**	**No – likely the opposite.** RhoBTB3 is considered a tumor suppressor; higher expression can correlate with better chemo response.	AML (acute leukemia) – **General chemotherapy**.	Clinical correlation.	[28-30]
**ZBTB41**	**No known association.**	–	– (No data)	–
**DCUN1D4**	**No known association.**	–	– (No data)	–
**EPN1**	**No known association.**	–	– (No direct data)	–
**ASH1L**	**Tentative link – overexpression in resistant states.** As an epigenetic activator, ASH1L is overexpressed in therapy-resistant cancers.	Prostate cancer – Castration-resistant (epigenetic dysregulation linked to hormone therapy resistance and progression); Acute leukemia – Maintains leukemogenic gene expression (e.g., HOXA9); potential therapeutic target for anti-leukemic activity.	*In vitro* and tumor data.	[31, 32]
**FNTB**	**No specific evidence.** (Overexpression could hypothetically sustain oncogenic RAS signaling, but no direct studies.)	–	– (No clear data)	–
**STX12**	**No known association.**	–	– (No data)	–

**MRFAP1**	**Yes**	Gastric cancer – MLN4924 (neddylation inhibitor)	Promotes chemoresistance via interaction with P27, stabilizing cell cycle progression	[33]

"No known association" indicates no experimental or clinical evidence currently links that gene's overexpression to drug resistance in cancer. For genes with positive associations, the cancer context, drugs, and evidence are described.

Table S5. Downregulated genes involved in autophagosome organization in SNU475 cells transfected with miR-142-3p.

SYMBOL	RANK IN GENE LIST	RANK METRIC SCORE	RUNNING ES	CORE ENRICHMENT
**STX12**	48	1.740268469	0.045600224	Yes
TP53INP2	98	1.482061625	0.08396765	Yes
ATG16L1	208	1.1973629	0.11097352	Yes
RAB1A	213	1.179813266	0.14352931	Yes
SNX30	228	1.163090348	0.17504539	Yes
**ULK1**	627	0.808376014	0.17462394	Yes
ATP13A2	630	0.807041168	0.19693577	Yes
SNX18	1032	0.658935666	0.19218901	Yes
RAB43	1035	0.658061028	0.21036085	Yes
PIP4K2B	1049	0.655122757	0.22781853	Yes
NUPR1	1111	0.637945473	0.24203882	Yes
AMBRA1	1134	0.631424904	0.25832045	Yes
SH3GLB1	1211	0.609076917	0.270876	Yes
SNX4	1291	0.589775145	0.28272265	Yes
MAP1LC3B2	1333	0.581213951	0.29651645	Yes
MAP1LC3A	1361	0.572930515	0.31088507	Yes
WDR45	1524	0.53998071	0.31657544	Yes
ATG2A	1586	0.528496683	0.32775423	Yes
ATG7	1616	0.522012889	0.34059292	Yes
ZFYVE26	1850	0.487843722	0.34075186	Yes
TRAF6	2012	0.46388346	0.34438503	Yes
ATG13	2019	0.462717503	0.3568985	Yes
PACS2	2057	0.458132029	0.36750197	Yes
ATG9A	2070	0.455351561	0.3794657	Yes
PIP4K2C	2331	0.421003103	0.37621468	Yes
TOM1	2395	0.410892785	0.3840104	Yes
MTOR	2451	0.405407429	0.39211372	Yes
PHF23	2485	0.400956005	0.40135834	Yes
RAB1C	2521	0.396475136	0.41036344	Yes
LRSAM1	2714	0.376529366	0.4097866	Yes
NSFL1C	2737	0.374114186	0.41891783	Yes
MFN2	2890	0.35994041	0.42018005	Yes
CTSD	2897	0.359301299	0.42981967	Yes

ATG14	2903	0.358853519	0.43950433	Yes
SMCR8	2977	0.351959854	0.44508734	Yes
UBQLN2	3014	0.347868621	0.45268422	Yes
ATP2A2	3074	0.340958238	0.45876655	Yes
RNF5	3142	0.335287452	0.46423128	Yes
MAP1LC3C	3155	0.333500147	0.4728089	Yes
GABARAP	3184	0.330599874	0.4803859	Yes
PIK3C2B	3304	0.318234921	0.4823867	Yes
CLN3	3492	0.300919592	0.47999626	Yes
MAP1LC3B	3561	0.295564264	0.48429963	Yes
FEZ2	3604	0.292958558	0.49002558	Yes
EFNB1	3867	0.273134828	0.48255047	Yes
EPHB2	3897	0.270199358	0.4883915	Yes
SEC22B	4177	0.251032799	0.47932467	Yes
MOAP1	4247	0.246472284	0.4822063	Yes
ULK3	4256	0.245468438	0.48856762	Yes
ARFIP2	4305	0.242145032	0.49253651	Yes
EMC6	4333	0.240752608	0.49767426	Yes
RAB1B	4504	0.229042232	0.49426395	Yes
ATG5	4548	0.226456463	0.4980844	Yes
ATG4D	4563	0.224942595	0.5035303	Yes
PIK3C3	4601	0.222379237	0.5075825	Yes
ATG4B	4622	0.221383244	0.51258445	Yes
PSEN1	4693	0.215919748	0.51455957	Yes
ATG3	4730	0.213070959	0.51841056	Yes
STBD1	4820	0.208445773	0.51908547	Yes
DNAJC16	4862	0.20580034	0.5224469	Yes
WDR45B	4880	0.204686537	0.5271574	Yes
IFT20	4902	0.203361779	0.53160113	Yes
PIKFYVE	4933	0.201166824	0.5354663	Yes
AP4M1	4957	0.199341491	0.5396833	Yes

Table S6. Downregulated genes involved in lysosomal function in SNU475 cells transfected with miR-142-3p.

SYMBOL	RANK IN GENE LIST	RANK METRIC SCORE	RUNNING ES	CORE ENRICHMENT
**SLC17A5**	13	2.357998371	0.079431325	Yes
CLTA	94	1.502696753	0.12592803	Yes
AP1S3	159	1.292705894	0.16620432	Yes
PPT2	424	0.93642652	0.18286785	Yes
SCARB2	643	0.799565315	0.19752231	Yes
ATP6V0A1	993	0.668929636	0.20020345	Yes
GM2A	1459	0.554777265	0.1923341	Yes
SLC11A2	1824	0.4914186	0.18811698	Yes
ARSA	1835	0.489744484	0.2041948	Yes
PLA2G15	1866	0.485828906	0.21898964	Yes

HGSNAT	1893	0.481869698	0.23387982	Yes
ACP2	1977	0.46809727	0.24502471	Yes
MFSD8	1980	0.467786729	0.26081583	Yes
ATP6V0D1	2192	0.438704014	0.2636024	Yes
AP3D1	2223	0.433921725	0.27663225	Yes
LAPTM4B	2385	0.412129283	0.28138974	Yes
CLTCL1	2434	0.407068998	0.2924717	Yes
CTSK	2487	0.400465012	0.30309913	Yes
AP3B1	2549	0.392863393	0.31295064	Yes
AP3S2	2564	0.39138785	0.3254541	Yes
CLTC	2580	0.389994979	0.33785266	Yes
CTSD	2897	0.359301299	0.33190274	Yes
NEU1	2979	0.351533145	0.3391991	Yes
ATP6V0C	3220	0.32819286	0.33656076	Yes
CLN3	3492	0.300919592	0.3312128	Yes
LGMN	3501	0.300322652	0.34096467	Yes
SMPD1	3730	0.283605427	0.33750013	Yes
CTSA	3831	0.275950104	0.3411341	Yes
AP3S1	3925	0.268322617	0.34491116	Yes
SGSH	4043	0.260490119	0.3470421	Yes
NAGPA	4264	0.24502027	0.3427255	Yes
CLTB	4275	0.244110763	0.35045102	Yes
CTSH	4278	0.244001985	0.3586328	Yes
GNS	4355	0.239379957	0.3624031	Yes
TPP1	4530	0.227243468	0.36012658	Yes
CTNS	4536	0.226991698	0.3675575	Yes
AP4B1	4593	0.223099455	0.371924	Yes
ATP6V1H	4607	0.222296715	0.37873536	Yes
AP1S1	4778	0.210450321	0.3761178	Yes
MANBA	4795	0.2097992	0.3823317	Yes
ABCA2	4835	0.207368493	0.38714066	Yes
FUCA1	4929	0.201417252	0.38864276	Yes
AP4M1	4957	0.199341491	0.39386868	Yes
LAPTM4A	5043	0.193480626	0.39556083	Yes
AP1M2	5097	0.190710902	0.39899853	Yes
TCIRG1	5204	0.185766697	0.39922106	Yes
AP3M1	5493	0.167988226	0.38837573	Yes
ARSG	5512	0.166841879	0.39301398	Yes
GNPTAB	5522	0.166168049	0.39814675	Yes
NPC2	5523	0.166112721	0.40379506	Yes
GGA1	5573	0.163066998	0.40652275	Yes
GAA	5575	0.162925556	0.4120052	Yes
IDS	5615	0.16031836	0.41521433	Yes
AP1B1	5621	0.160059005	0.42036933	Yes
NAGA	5660	0.158111453	0.42356092	Yes

References

1. Hidalgo-Sastre, A., et al., *Loss of Wasl improves pancreatic cancer outcome.* JCI Insight, 2020. **5**(10).
2. Salvi, A. and T. Thanabalu, *Expression of N-WASP is regulated by HiF1α through the hypoxia response element in the N-WASP promoter.* Biochemistry and Biophysics Reports, 2017. **9**: p. 13-21.
3. Zimmer, N., et al., *GARP as a Therapeutic Target for the Modulation of Regulatory T Cells in Cancer and Autoimmunity.* Front Immunol, 2022. **13**: p. 928450.
4. Yu, B.B., et al., *Cofilin-2 Acts as a Marker for Predicting Radiotherapy Response and Is a Potential Therapeutic Target in Nasopharyngeal Carcinoma.* Med Sci Monit, 2018. **24**: p. 2317-2329.
5. Wo, Q., et al., *Long noncoding RNA SOX2-OT facilitates prostate cancer cell proliferation and migration via miR-369-3p/CFL2 axis.* Biochemical and Biophysical Research Communications, 2019. **520**(3): p. 586-593.
6. Li, H., et al., *Nuclear orphan receptor NR2F6 confers cisplatin resistance in epithelial ovarian cancer cells by activating the Notch3 signaling pathway.* Int J Cancer, 2019. **145**(7): p. 1921-1934.
7. Kim, H., et al., *Melanoma-intrinsic NR2F6 activity regulates antitumor immunity.* Science Advances, 2023. **9**(27): p. eadf6621.
8. Dougherty, M.I., et al., *PRAS40 Phosphorylation Correlates with Insulin-Like Growth Factor-1 Receptor-Induced Resistance to Epidermal Growth Factor Receptor Inhibition in Head and Neck Cancer Cells.* Mol Cancer Res, 2020. **18**(9): p. 1392-1401.
9. Xiu, L., et al., *High expression of RARG accelerates ovarian cancer progression by regulating cell proliferation.* Front Oncol, 2022. **12**: p. 1063031.
10. Chen, X., et al., *A novel NPM1-RARG-NPM1 chimeric fusion in acute myeloid leukaemia resembling acute promyelocytic leukaemia but resistant to all-trans retinoic acid and arsenic trioxide.* Br J Cancer, 2019. **120**(11): p. 1023-1025.
11. Pan, D., et al., *Radiation induces premature chromatid separation via the miR-142-3p/Bod1 pathway in carcinoma cells.* Oncotarget, 2016. **7**(37).
12. Shi, Y., et al., *Cholesterol-enriched membrane micro-domaindeficiency induces doxorubicin resistancevia promoting autophagy in breast cancer.* Mol Ther Oncolytics, 2021. **23**: p. 311-329.
13. Zhao, Y., et al., *CCAAT enhancer binding protein delta activates vesicle associated membrane protein 3 transcription to enhance chemoresistance and extracellular PD-L1 expression in triple-negative breast cancer.* Journal of Experimental & Clinical Cancer Research, 2024. **43**(1): p. 115.
14. Zhai, K., et al., *Overexpression of TWF1 promotes lung adenocarcinoma progression and is associated with poor prognosis in cancer patients through the MMP1 signaling pathway.* J Thorac Dis, 2023. **15**(5): p. 2644-2658.
15. Bockhorn, J., et al., *MicroRNA-30c inhibits human breast tumour chemotherapy resistance by regulating TWF1 and IL-11.* Nat Commun, 2013. **4**: p. 1393.
16. Wang, Y., et al., *TWF1 induces autophagy and accelerates malignant phenotype in lung adenocarcinoma via inhibiting the cAMP signaling pathway.* Faseb j, 2023. **37**(7): p. e23051.

17. Sekulic, A., et al., *Loss of inositol polyphosphate 5-phosphatase is an early event in development of cutaneous squamous cell carcinoma.* Cancer Prev Res (Phila), 2010. **3**(10): p. 1277-83.
18. Sato, H., et al., *SRC Family Kinase Inhibition Targets YES1 and YAP1 as Primary Drivers of Lung Cancer and as Mediators of Acquired Resistance to ALK and Epidermal Growth Factor Receptor Inhibitors.* JCO Precis Oncol, 2022. **6**: p. e2200088.
19. Garmendia, I., et al., *YES1: A Novel Therapeutic Target and Biomarker in Cancer.* Mol Cancer Ther, 2022. **21**(9): p. 1371-1380.
20. Zhou, H., et al., *Role of YES1 signaling in tumor therapy resistance.* Cancer Innov, 2023. **2**(3): p. 210-218.
21. Shu, Y., et al., *FOS-Mediated PLCB1 Induces Radioresistance and Weakens the Antitumor Effects of CD8(+) T Cells in Triple-Negative Breast Cancer.* Mol Carcinog, 2025. **64**(1): p. 162-175.
22. Liang, S., et al., *A PLCB1-PI3K-AKT Signaling Axis Activates EMT to Promote Cholangiocarcinoma Progression.* Cancer Res, 2021. **81**(23): p. 5889-5903.
23. Ma, K., et al., *Increased oxygen stimulation promotes chemoresistance and phenotype shifting through PLCB1 in gliomas.* Drug Resist Updat, 2024. **76**: p. 101113.
24. Wang, W.W., et al., *Integrin beta-8 (ITGB8) silencing reverses gefitinib resistance of human hepatic cancer HepG2/G cell line.* Int J Clin Exp Med, 2015. **8**(2): p. 3063-71.
25. Liu, S., et al., *Integrin β8 facilitates tumor growth and drug resistance through a Y-box binding protein 1-dependent signaling pathway in bladder cancer.* Cancer Sci, 2020. **111**(7): p. 2423-2430.
26. Ma, C., et al., *PAFAH1B2 is a HIF1a target gene and promotes metastasis in pancreatic cancer.* Biochemical and Biophysical Research Communications, 2018. **501**(3): p. 654-660.
27. He, Y., et al., *Platelet-activating factor acetyl hydrolase IB2 dysregulated cell proliferation in ovarian cancer.* Cancer Cell Int, 2021. **21**(1): p. 697.
28. Ji, W. and F. Rivero, *Atypical Rho GTPases of the RhoBTB Subfamily: Roles in Vesicle Trafficking and Tumorigenesis.* Cells, 2016. **5**(2).
29. Yang, S.H., et al., *High Expression of RhoBTB3 Predicts Favorable Chemothrapy Outcomes in non-M3 Acute Myeloid Leukemia.* J Cancer, 2021. **12**(14): p. 4229-4239.
30. Zhang, C.-S., et al., *RHOBTB3 promotes proteasomal degradation of HIFα through facilitating hydroxylation and suppresses the Warburg effect.* Cell Research, 2015. **25**(9): p. 1025-1042.
31. Yu, M., et al., *Structural insight into ASH1L PHD finger recognizing methylated histone H3K4 and promoting cell growth in prostate cancer.* Front Oncol, 2022. **12**: p. 906807.
32. Rogawski, D.S., et al., *Discovery of first-in-class inhibitors of ASH1L histone methyltransferase with anti-leukemic activity.* Nature Communications, 2021. **12**(1): p. 2792.
33. Hu, L., et al., *MRFAP1 plays a protective role in neddylation inhibitor MLN4924-mediated gastric cancer cell death.* Eur Rev Med Pharmacol Sci, 2018. **22**(23): p. 8273-8280.
